# Visualization and Exploration of Conserved Regulatory Modules Using ReXSpecies 2

**DOI:** 10.1186/1471-2148-11-267

**Published:** 2011-09-24

**Authors:** Stephan Struckmann, Daniel Esch, Hans Schöler, Georg Fuellen

**Affiliations:** 1 University of Rostock, Institute for Biostatistics and Informatics in Medicine and Ageing Research, Heydemannstrasse 8, 18057 Rostock, Germany; 2 E.M.A. University of Greifswald, Institute for Mathematics and Computer Science, Rathenaustrasse 47, 17487 Greifswald, Germany; 3 Department of Cell and Developmental Biology, Max Planck Institute for Molecular Biomedicine, Röntgenstrasse 20, 48149 Münster, Germany; 4 Medical Faculty, University of Münster, Domagkstrasse 3, 48149 Münster, Germany

## Abstract

**Background:**

The prediction of transcription factor binding sites is difficult for many reasons. Thus, filtering methods are needed to enrich for biologically relevant (true positive) matches in the large amount of computational predictions that are frequently generated from promoter sequences.

**Results:**

ReXSpecies 2 filters predictions of transcription factor binding sites and generates a set of figures displaying them in evolutionary context. More specifically, it uses position specific scoring matrices to search for motifs that specify transcription factor binding sites. It removes redundant matches and filters the remaining matches by the phylogenetic group that the matrices belong to. It then identifies potential transcriptional modules, and generates figures that highlight such modules, taking evolution into consideration. Module formation, scoring by evolutionary criteria and visual clues reduce the amount of predictions to a manageable scale. Identification of transcription factor binding sites of particular functional importance is left to expert filtering. ReXSpecies 2 interacts with genome browsers to enable scientists to filter predictions together with other sequence-related data.

**Conclusions:**

Based on ReXSpecies 2, we derive plausible hypotheses about the regulation of pluripotency. Our tool is designed to analyze transcription factor binding site predictions considering their common pattern of occurrence, highlighting their evolutionary history.

## Background

The investigation of gene regulation in general, and transcription factor binding in particular, is a challenging task [1-3], and many data sources should be considered. In silico predictions based on position specific scoring matrices (describing binding motifs) suffer from many matches. These matches may be false positives, since they only rely on the primary structure of a sequence. For instance, the spatial structure and accessibility of DNA, which is not only defined by its sequence, is important when considering transcription factor binding. Hence, predictions made using only the primary structure of a sequence are usually not presumed to be very specific.

One may expect that similarity between a base sequence and a known binding motif is a necessary condition for binding, but it is neither a necessary, nor a sufficient one. On one hand, transcription factors can bind sequences that are quite dissimilar from their known binding motif. On the other hand, other effects (such as spatial DNA structure) can prevent the transcription factor from binding, even if the binding motif is matching. Nevertheless, to arrive at a binary binding/non-binding statement, predictions with a sequence-based similarity score below an arbitrary threshold are usually discarded. A binary decision is of course problematic, since binding and functionality are both better reflected by a gradual scale. In vitro analyses may likewise not be reliable. That is, even if transcription factor binding is measured, this may be an experimental artifact and does not necessarily imply regulatory effects. No method or algorithm based on sequence data alone can predict regulatory function of individual transcription factor binding sites, even if binding has been demonstrated in vivo.

To make the best of this situation, we and others proposed to integrate data from different sources [4,5]. Apart from computational predictions, curated data and gene expression experiments can be considered; some are available from genome browsers such as the UCSC browser [6]. However, such data is usually *not *available. Then again, conservation data on the sequence level is very often available, and conservation of transcriptional modules [5] can help to improve hypotheses about transcriptional regulation. Based on this premise, using the conservation of binding sites between species, we developed a tool called ReXSpecies [7,8] for the identification of transcriptional modules, and of their gain/loss patterns in evolution.

In the following, a *transcriptional module *is defined as *a set of transcription factor binding sites*, and *a gain/loss pattern *is defined as *a transcriptional module, for which we find a gain or loss in evolution*, see Implementation. Please note that the term *transcriptional module *as defined here is closely related to the term *regulatory module*. In turn, the term *regulatory module *has three distinct definitions in the literature. A *regulatory module *is defined either ***(a) **as a sequence region that contains regulatory functions with no particular reference to transcription factor binding sites, or **(b) **as a particular transcription factor binding site combination that has one defined transcriptional function, or **(c) **as a sequence region that contains regulatory functions, including transcription factor binding site combinations that may separately or simultaneously have one or more transcriptional functions*. ReXSpecies aims to find regulatory modules in the sense of definition **(c)**. It highlights regions in sequences as potentially functional and proposes transcriptional modules, i.e. sets of transcription factor binding sites providing one or more of such functions.

Here, we present version 2.0 of ReXSpecies, which offers an array of new features, and has been improved in terms of graphical visualization, interactivity, and usability. It provides full automation; the only input needed is a gene name, or the coordinates of a promoter region. In case of a gene name, a genomic region upstream of the transcription start site is taken, based on UCSC [9] data. Starting from a precomputed selection of homologous regulatory regions available via UCSC [9], ReXSpecies 2 automatically searches for transcription factor binding sites therein, using position specific scoring matrices and the PoSSuM tool [10]. Further, it generates novel hypotheses about modules and the gain/loss patterns associated with these. Such groups of predicted transcription factor binding sites are relevant for the study of gene regulation; their evolutionary conservation and history can now be visualized by means of the following figures:

1. An annotated alignment, combining sequence and binding site information,

2. An annotated phylogenetic tree, including ancestral states estimated for the transcription factor binding sites found in today's species,

3. A combination of alignment and tree,

4. Interactive web pages of 1-3,

5. Interactive genome browser web pages (e.g. UCSC [6]). In particular, ReXSpecies 2.0 can now write BED file format [11] that can be visualized using the UCSC genome browser [6] and the EnsEMBL genome browser [12].

See also Additional File 1, Part II for an overview of all figure types.

### Interpretation of predicted transcription factor binding sites - lots of false positives or lots of ubiquitous transcription?

As methods for the investigation of gene regulation neglect some aspects of binding and frequently generate a large amount of predictions, a high false positive rate is usually assumed. However, an alternative to declaring that most predicted transcription factor binding sites are false positives is motivated by the concept of "ubiquitous transcription" [13]. In general, ubiquitous transcription refers to the observation that virtually every nucleotide is transcribed [13,14], even though often at low levels, and usually with negligible biological effect. The biological effect may in turn depend on the biological context. Ubiquitous transcription can be explained by a utilization of binding sites by transcription factors that is much higher than usually estimated. Such binding would be highly context-dependent; in particular it may depend on transcription factor abundance, and on epigenetic influences that modify DNA accessibility. If the regulatory regions of specific genes are studied, as will be the case here, high utilization of binding sites means that many predicted binding sites and corresponding modules are functional, even though many of them are only functional in specific, often negligible contexts.

In this paper, we cannot and do not need to prove or disprove ubiquitous transcription. *However, the actual amount of ubiquitous transcription has implications for the interpretation of the results we find*. The more ubiquitous transcription there is, the more likely the predicted binding sites and corresponding modules are functional, and the closer the findings we report below are a true reflection of reality. In the remainder of this paper, our interpretation will be on the conservative side, and we will write about the "large amount of false positives" in the usual fashion. In the extreme case, almost every predicted binding site may be considered "false positive". We suggest, however, that binding sites found by ReXSpecies, which are included in an evolutionarily conserved module and which are gained or lost together, have a good chance of being "true positive". In the Results and Discussion section, we wish to demonstrate that there is some evidence for our suggestion provided by the plausibility of many of the modules that we detect.

### Other approaches to filter transcription factor binding sites using evolutionary conservation

As discussed, we assume that there are many putatively false positive predicted transcription factor binding sites found as matches of position specific scoring matrices in eukaryotic genomes (see also [7]). In our case, predictions will be filtered by taking binding sites forming a module that is conserved in evolutionary time. A critical point for filtering by evolutionary conservation is to identify homology. Such homology should not just hold for the base sequence. Instead, evolutionary constraints also apply to binding effects, which depend on the spatial structure and accessibility of the DNA and on binding affinities (also weak binding) of transcription factor molecules to the DNA, which classical alignments based on sequence (Smith-Waterman, BLAST) fail to consider. Moreover, functional equivalence of different sub-sequences may be retained although the sequence itself changes heavily.

To overcome some of these difficulties, two approaches are possible in principle. One way is to first search for transcription factor binding sites, and to afterwards identify homology (*alignment free methods*). The other way is to start with finding homologous sequences and their alignment, and then to search for transcription factor binding sites therein (*phylogenetic footprinting*). In each case, the first step is crucial.

• Finding transcription factor binding sites first *(alignment free) *means that it is not possible to use evolutionary conservation for that step, and a decline in specificity may be the result.

• If analyses start generating an alignment *(phylogenetic footprinting)*, the result, i.e. statements about homology on the sequence level, given by the alignment, may be of low quality. During the alignment step, no knowledge about the putative transcription factor binding sites found in the sequences can then be used, so their possible homology cannot be taken into account. For example, some binding sites may inadvertantly be torn apart by the alignment in one of the sequences, because they are not considered during the alignment step.

One *alignment-free method *is the Regulatory Region Score (RRS) proposed by Koohy et al. [15]. It combines gene expression related features with sequence features and then calculates average scores for sequences to be bound by certain transcription factors. It afterwards generates a list of functionally homologous sequences.

Unlike the RRS method, ReXSpecies 2.0 belongs to the *phylogenetic footprinting *methods, generating an alignment first. It does not generate de-novo *transcription factor binding site motifs*, but instead it uses libraries of known binding site motifs, and it subsequently generates de-novo *modules *of transcription factor binding sites. Manke et al. [16] published such a method that finds transcription factor binding site modules in conserved regulatory sequences. It is based on gene expression data and gene function (gene ontology [17]) data and their algorithm does not directly consider a phylogeny, but evolutionary conservation is used. Similarly, MCAST [18] allows one to search for clusters of motif matches, but without using phylogenic information. GibbsModule [19] and PhyME [20] provide *de-novo *detection of motifs and transcriptional modules constituted by such motifs. Compared to these two tools, ReXSpecies 2.0 uses validated libraries of position specific scoring matrices [21,22] for finding transcription factor binding sites and searches for sets of potentially cooperating transcription factor binding sites, using their phylogenetic conservation.

Other important frameworks used to analyze transcriptional regulation are Genomatix [23] with its FrameWorker module, Transfac [24] and Mapper [25]. Except for Mapper, these tools are only commercially available. Mapper and Transfac do not search for modules, but for transcription factor binding sites only. One feature of Mapper is that it provides a user interface to MEME [26], a tool to generate *de novo *transcription factor binding site models. Genomatix FrameWorker does not reconstruct ancestral states, but searches in a set of sequences for common patterns to detect modules.

Many of the approaches listed above only generate lists of putative modules but lack the ability to render sophisticated figures. When these approaches generate figures (Genomatix) or interact with genome browsers (Mapper), they only show aligned sequences where glyphs (squares/rectangles) below these sequences represent the predictions. They fail to show the evolutionary history of single transcription factor binding sites or CRMs. ReXSpecies 2.0 provides some novel figure types (see "Results and Discussion: Output Files generated by ReXSpecies") that were designed to analyze transcriptional regulation, including its evolution.

## Results and Discussion

### Input for ReXSpecies 2.0

**Figure 1:**
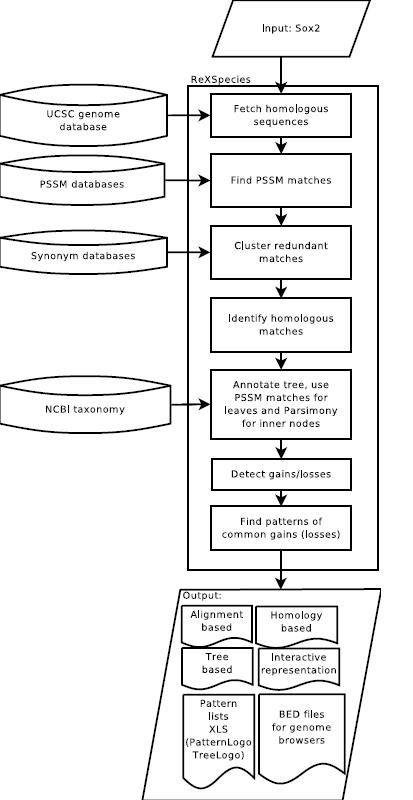
**Flowchart of a ReXSpecies 2.0 analysis**. The steps as shown are executed automatically upon user input. Necessary input are only the gene name or the genome coordinates of the region to analyze. *Optionally*, most steps can be configured further: other PSSMs may be selected, the thresholds for the PSSM search tool can be set up, clustering and homology detection can be adjusted, the species tree to use can be selected, the patterns can be filtered based on different criteria, and the figures can be configured (size, format, hide/show elements, pattern highlighting). Further data curation can then be done manually.

Once logged in to the ReXSpecies web-server to start a new analysis, only genome coordinates (either mouse or human) or a gene name have to be entered. Additionally, the set of position specific scoring matrices to use (JASPAR [21] matrices are available in ReXSpecies, other matrices can be uploaded by the user) may be changed and the parameters for the different stages/steps (see Figure 1) of the analysis may be adjusted.

### Workflow of ReXSpecies 2.0

**Figure 2:**
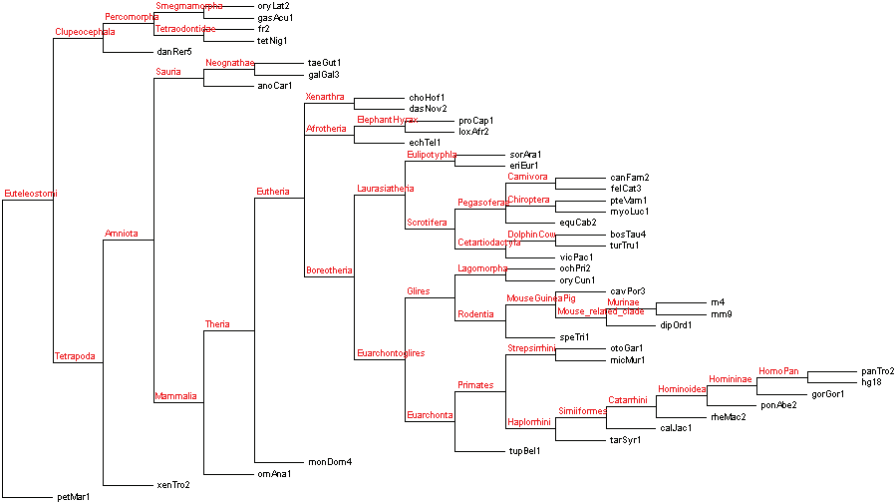
**The taxonomic tree used by ReXSpecies 2.0 by default**. The tree is based on the taxonomic trees published in [27-29]. The species codes at the leaves are the standard UCSC species codes.

After starting an analysis, ReXSpecies 2.0 first supplements the data with a set of regulatory regions from other species homologous to the selected one. This is done using the multiz track of the UCSC genome database [6,9]. In the next step (see Figure 1), sequence based predictions for transcription factor binding sites are generated by PoSSuM [10], in particular based on the JASPAR position specific scoring matrix database [21]. In the following two steps (see Figure 1), these predictions are aligned and annotated, such that redundant matches are clustered and homologous predictions are detected. This generates a table of predictions for each species. Then, in the 5th step (Figure 1), the nodes of the phylogenetic species tree for the species under investigation [27-29] (Figure 2) are labelled using the table of predictions and Fitch parsimony [30] reconstruction for the ancestral states. The edges of this tree are then, in the 6th step, annotated with all gains and losses of transcription factor binding sites based on the node annotations generated in the step before. Finally, in the set of predicted gains and losses, ReXSpecies 2.0 searches for gain/loss patterns of modules and generates the output files as described next.

### Output Files generated by ReXSpecies 2.0

**Figure 3:**
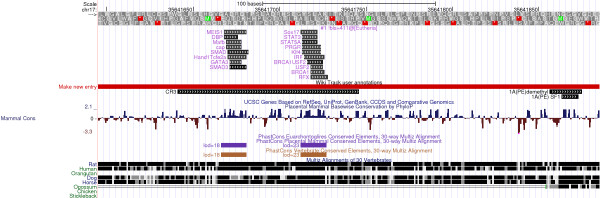
**CR3 pattern at UCSC**. The pattern found in the murine CR3 region at the UCSC genome browser. The pattern is shown as one track, more patterns would be in a new track. The tracks are sorted by branch length score [37], which is shown in the track description. The color and the number in the track description are unique for each pattern, the ancestral species predicted to be the origins of the pattern are also given in the track description. (If all transcription factor binding sites of a pattern are predicted for mouse, this is denoted using an exclamation mark.) If a pattern does not occur for mouse at all, it is not shown in the UCSC figure. The color intensity of the glyphs (squares/rectangles) that represent the binding sites reflects the matrix similarity score. This score is based on the position specific scoring matrix match that yielded the murine transcription factor binding site prediction. Below the prediction tracks, the wiki track is shown. This track contains annotations that we have listed in [5], see also Table 1. The wiki track entries are linked with the PubMed [42] entries of the papers that published the corresponding transcription factor binding site. At the bottom of the figure, sequence conservation is shown. We analyzed the PhastCons [43] most conserved elements only; also we did not analyze the untranslated regions (UTRs) of genes. The very first track lists the nucleotide bases; stop codons (*) in the "amino acid track" indicate that the region is non-coding.

**Figure 4:**
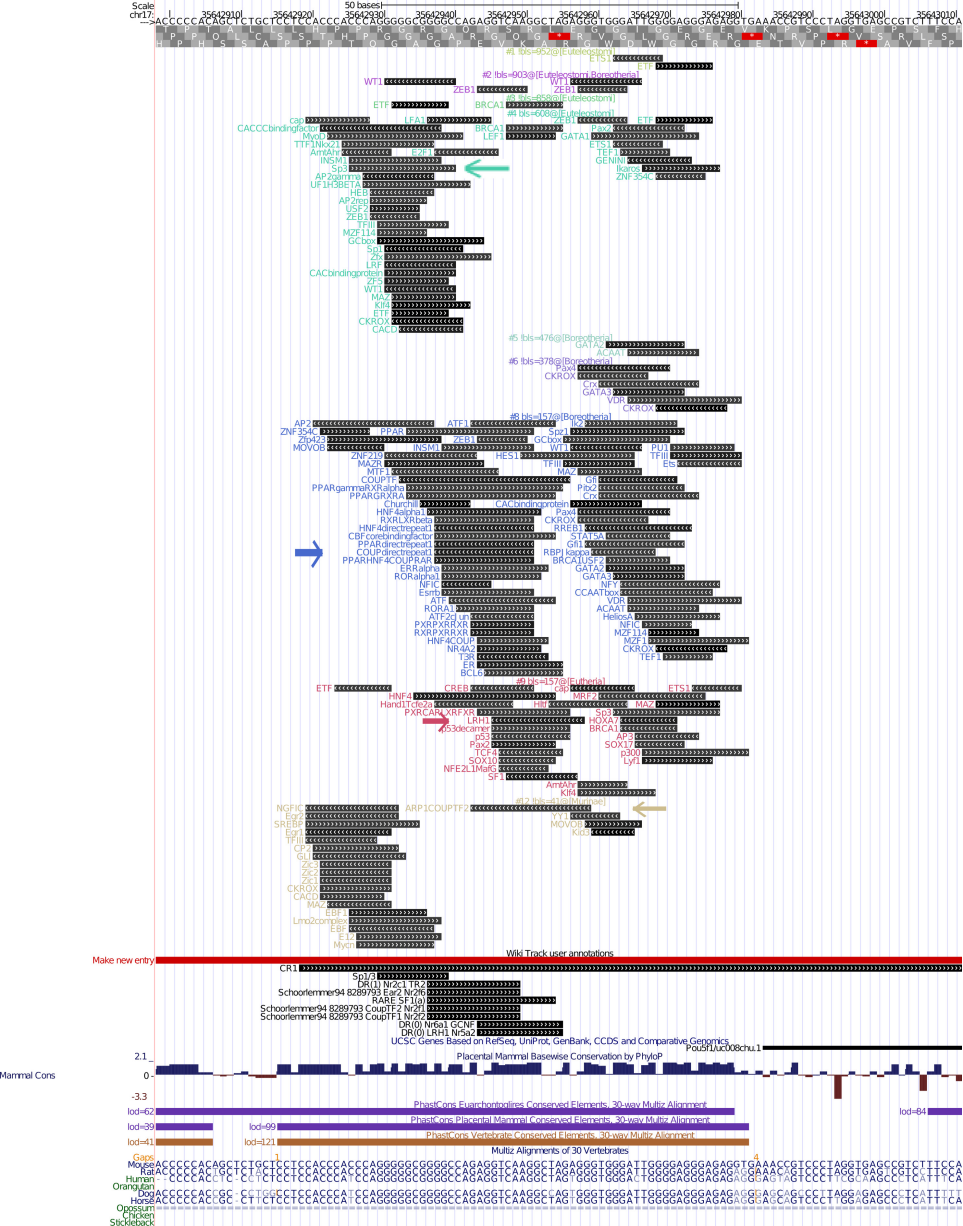
**CR1 patterns at UCSC**. The patterns found in the murine CR1 region at the UCSC genome browser. See Figure 3 for further explanations.

**Figure 5:**
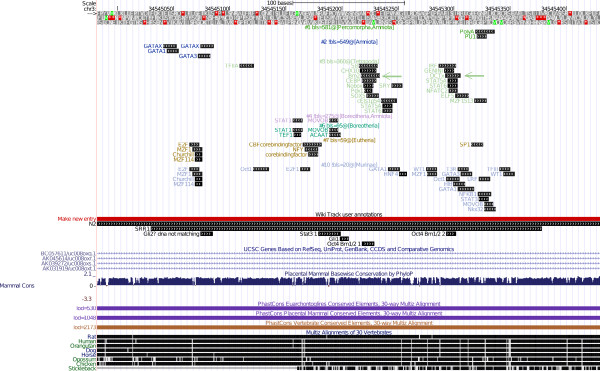
**SRR1 patterns at UCSC**. The patterns found in the murine SRR1 region at the UCSC genome browser. See Figure 3 for further explanations.

**Figure 6:**
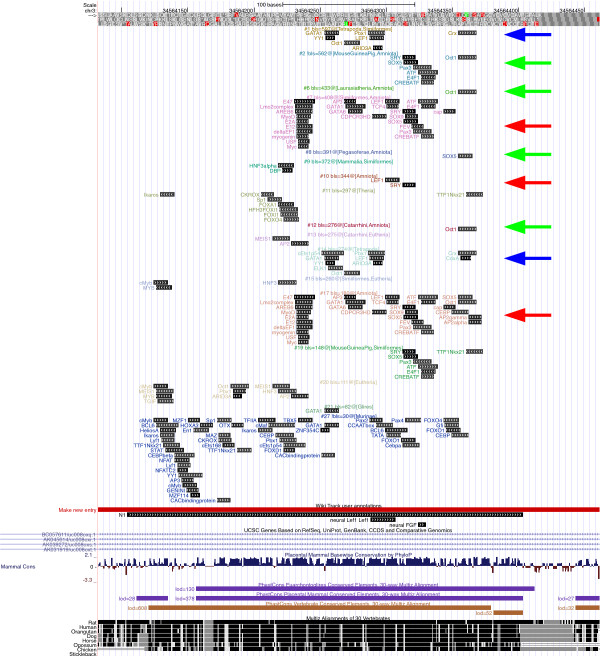
**N1 patterns at UCSC**. The patterns found in the murine N1 region at the UCSC genome browser. See Figure 3 for further explanations.

**Figure 7:**
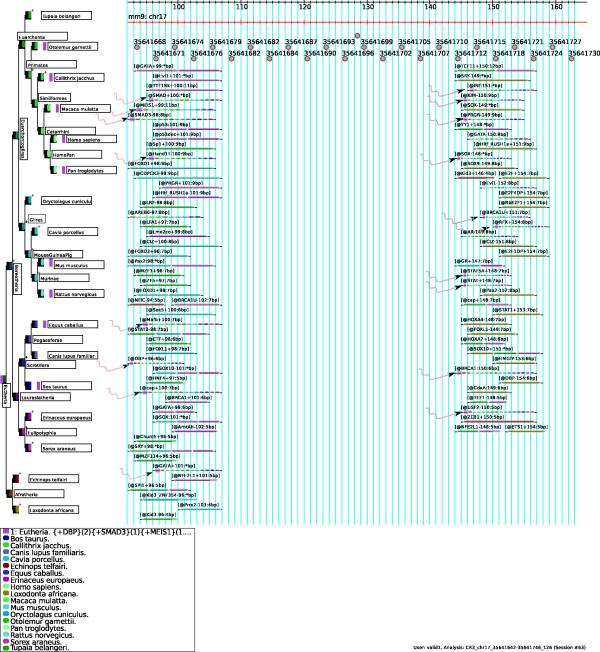
**CR3 pattern, Homology-based figure**. The pattern and the transcription factor binding sites found in the murine CR3 region. The various components of the homology based figure are described in the main text, under the heading "Legend of the homology based figure".

ReXSpecies 2.0 generates different files, especially figures that allow users to visualize regulatory sequences and that highlight evolutionary conserved modules. All figures can be exported in PDF format. Depending on a file's content (table or figure), it can also be exported in different spreadsheet formats (ODF, XLS), or in common graphic formats (PNG, TIFF, BMP, SVG, EPS, PS). Additionally, ReXSpecies can write BED files [11]. Such BED files (optionally extended BED files with STRING [31,32] and iHOP [33,34] information about the transcription factor proteins) are input files for genome browsers such as UCSC [6,35] or EnsEMBL [12,36]. The genome browser can then be used to generate figures of the transcription factor binding site predictions made by ReXSpecies, with extended annotations available via the user interface of the genome browser as clickable links. (See Figures 3, 4, 5, 6 and Additional files 2, 3, 4, and 5.) ReXSpecies 2.0 can also generate figures that show an annotated alignment with color-coded species, giving an overview of the annotated sequences (See Figure 7, and Additional Files 6, 7, 8). Another type of output file is a table (available as Excel sheet, as OpenDocument sheet or as PDF) that lists all modules with gain/loss patterns (See Additional Files 9, 10, 11, 12, 13, 14, 15, and 16). All output files are systematically presented in Additional File 1, Part II.

#### BED files, and UCSC visualization

Using UCSC [6] and EnsEMBL [12,36], custom annotation tracks may be uploaded in BED (Browser Extensible Data) format [11]. Therefore, we implemented export of results generated by ReXSpecies 2.0 as BED files [11]. These files can be uploaded to the UCSC genome browser and to EnsEMBL. UCSC can also directly be opened from ReXSpecies 2.0 with the BED file already uploaded, enabling scientists to analyze ReXSpecies 2.0 predictions in a familiar manner together with other features as proposed in [5]. (See Figure 3, 4, 5, 6 and Additional files 2, 3, 4 and 5. ReXSpecies 2.0 can also write a "BED detail format", so that information about each transcription factor acquired from the STRING database [31,32] and the iHOP database [33,34] is directly available through the web interface of UCSC.

As specified by the user, BED files may contain either modules of transcription factor binding sites, or transcription factor binding sites with a branch length score (BLS, [37]) above a given threshold. Given a phylogenetic species tree annotated with binding site predictions, the branch length score corresponds to the summation of all branch lengths (from UCSC multiz track [28]) of the subtree, in which for all leaves the prediction is annotated. Thus, the BED files contain either transcription factor binding sites as parts of modules, or highly conserved transcription factor binding sites filtered and ranked by their individual branch length score.

#### Annotated Alignment (Homology-based)

In genome browsers, figures are generated in a sequence-centered way and annotations are usually plotted below the sequence. A complementary sequence-centered figure is generated by ReXSpecies since 1.0 visualizing the transcription factor binding sites species-by-species (see Additional File 1, part II). This figure is called *alignment-based figure*. Examples are provided in Additional Files 17, 18, 19 and 20. However, because we usually consider homologous sequences from many species, which often contain many predictions, we will generate very large images if we consider all species in the alignment separately. To consider all species simultaneously, ReXSpecies (since version 1.0) clusters homologous predictions, and the size of the figure can thus be reduced to fewer glyphs (squares/rectangles) by eliminating redundant ones. Only one glyph is then used for each transcription factor binding site prediction, instead of many different ones for each prediction in each species. To generate such figures, we implemented a *homology-based *rendering in ReXSpecies 2.0 where each prediction is shown only once, as in Figure 7. For each transcription factor binding site prediction, all species in which homologous transcription factor binding sites occur are color coded. In Figure 7, the species tree is plotted to the left of the predictions and its nodes are colored the same way as the predictions are.

We developed a color assignment algorithm that assigns similar colors to closely related species and different colors to distant species. This algorithm implements the following principles:

• The color hue of the nodes follows a gradient along the leaves of the tree, so that evolutionary distant nodes are assigned dissimilar colors while evolutionary close nodes are similarly colored.

• Nodes close to the root get darker colors than nodes close to the leaves.

Homology-based figures emphasize highly conserved predictions in a natural way since they are rendered in many colors, because they are present in many species. (Such highly conserved homologs are also reflected by high branch length scores.) Also, it is easy to find predictions that are present in two distant regions of the species tree, because for each region, a block of similar colors is shown. Such predictions present in two distant regions often form a gain/loss pattern.

As in all types of figure (alignment- and homology-based figures), gain/loss patterns can be highlighted using the pattern color option. If activated, colors are assigned to patterns, and the pattern colors are shown in the left half *of each prediction box in the alignment*, while node-(gradient)-colors of the transcription factor binding site prediction (with colors denoting the species) are plotted to the right of the pattern colors. The nodes are then split in half, where the left half shows the pattern colors, and the right half shows the node (gradient) colors. Examples for homology-based figures are Figure 7 and Additional Files 6, 7, 8.

#### Legend of the homology based figure

In Figure 7, the pattern and the transcription factor binding sites found in the murine CR3 region are visualized species by species. The leaves of the species tree consist of colored boxes that are split into a left and a right half. On the left, all patterns that are predicted to be gained at this node by parsimony are indicated by color (see the root node); on the right, another color indicates the species corresponding to the node. In case of CR3, only one pattern is found, and it is gained at the root; hence we see only the dark pink color for this one pattern at the root. As explained above, species are indicated using another color scheme, going from green (top) to brown (bottom). A plus "+" sign next to the box denotes that a node gains transcription factor binding sites predicted by parsimony. Between the box just discussed, and the label with the species name, another box (or set of boxes) indicates those patterns, whose transcription factor binding sites all are predicted to exist for the species in question. In case of CR3, only one pattern is found, hence we see only the dark pink boxes for this one pattern. On top, alignment coordinates and genome coordinates of the reference genome (mm9 in this case) are shown. The transcription factor binding sites (i.e., usually, clusters of transcription factor binding sites) are labelled using their cluster name that contains the name of the transcription factor, the strand (if identical for all transcription factor binding sites clustered and all homologs), the (middle) position in alignment coordinates and the length of the transcription factor binding site in base pairs (without gaps), if identical for all clustered transcription factor binding sites and homologs. The transcription factor binding sites have colored overlays that represent the species where homologs of that transcription factor binding site are predicted. The thicker part of these overlays (pointed to by curvy pink arrows) indicates the patterns (glyphs with sharp edges), for which the transcription factor binding site is predicted by parsimony. The thin part indicates extant species (leaves), for which the transcription factor binding site is predicted using position specific scoring matrices and clustering. In the legend below the figure, all leaf/species and all pattern colors are explained. To keep the figures readable, by default, only the first 10 patterns (ordered by branch length score) are shown in the homology-based figures.

#### Table with all Details about Patterns

The most extensive output is the full list of gain/loss patterns of transcriptional modules. Each pattern is shown with its branch length score, the nodes in the tree where the gain/loss occurred, its transcription factor binding sites, and two logos:

• The pattern logo (PatternLogo) shows the pattern as it would occur in the alignment-based figure. That is, it shows a small alignment-based zoom-in where only the transcription factor binding sites consisting of the pattern are drawn.

• The tree logo (TreeLogo) shows the species tree. In this tree, the ancestral inner gain/loss nodes of the pattern as found by parsimony (as described in the implementation section below) are highlighted in the pattern color. Furthermore, all leaves of extant species, where the full pattern is present, are drawn in that color. The TreeLogo is strongly related to the branch length score. More specifically, the branch length score increases when there are more highlighted leaves and when the evolutionary distance separating the leaves is greater.

The pattern table can also be downloaded in Excel [38] or OpenOffice calc OpenDocument format [39], as a PDF or as an XML file, which could be processed further, using XSL transformation [40].

A long version of the pattern table includes sequence logos [41] for each sequence motif that is part of the pattern. Furthermore, direct links to different databases are provided, linking to

• the NCBI EntrezGene [42] entries for the genes that code for the transcription factors,

• the STRING [31,32] network of all transcription factors and the target gene of the regulatory sequence,

• a Google search for the transcription factors of the pattern and the target gene of the regulatory sequence,

• iHOP [33,34] searches for the transcription factor names, especially for those that are not mapped to any gene identifier.

Each match can easily be found in the figures generated by ReXSpecies (since 1.0) using a JavaScript function that highlights the corresponding elements, using the ReXSpecies web site. Pattern tables can be found in the supplement as Additional Files 9, 10, 11, 12, 13, 14, 15 and 16.

### Insights into the regulation of two of the core genes of pluripotency, Pou5f1 and Sox2

In the following, we will exemplify the use of ReXSpecies 2.0 by four case studies. We used position specific scoring matrices from two databases [21,22] to analyze the so-called "most conserved elements" (PhastCons [43]) of known regulatory sequences of pluripotency related genes. For the following case studies, we have chosen the CR1 (without the untranslated region of the overlapping first exon) and the CR3 of the murine *Pou5f1 *gene [44]. The CR1 is found at chr17:35,642,919-35,643,044, and CR3 at chr17:35,641,642-35,641,746 in version 9 of the mouse genome. Further, we analyzed the N1 and SRR1 [45,46] regions of the murine *Sox2 *gene. The N1 region is found at chr3:34,564,105-34,564,403 and the SRR1 region at chr3:34,545,031-34,545,378; it is part of the N2 region [45,46]. ReXSpecies 2.0 was used to remove duplicate matches, to find putative homologous matches, and to remove implausible matches such as plant specific transcription factor binding sites in mammals. Further, it was used to generate figures to visualize transcription factor binding in an evolutionary context and to highlight interesting evolutionary patterns.

Here, we present ReXSpecies 2 figures for all above mentioned regions and formulate hypotheses about their biological function. We will discuss these hypotheses in the context of what is known about these regions. Therefore, Table 1 includes all transcription factors listed in [5] that are known to bind to the regions investigated here, indicating which of these are available to ReXSpecies 2.0 based on the JASPAR/Transfac [21,22] libraries it uses. Table 1 is only used to discuss ReXSpecies results; there is no initial expert-guided restriction on the transcription factor binding sites considered by ReXSpecies.

**Table 1:** Transcription factor binding sites from our wiki track annotation [5] of the regulatory regions investigated here

Transcription factor	JASPAR	Transfac	Region
Sp1	+	+	CR1
Sp3	-	+	CR1
TR2	-	-	CR1
Nr2c1	-	-	CR1
Ear2	-	-	CR1
Nr2f6	-	-	CR1
RARE	-	-	CR1
SF1	-	+	CR1
COUP-TF2	-	+	CR1
Nr2f1	+	+	CR1
COUP-TF1	+	+	CR1
Nr2f2	-	+	CR1
Nr6a1	-	-	CR1
GCNF	-	+	CR1
LRH-1	-	+	CR1
Nr5a2	-	-	CR1
Fgf	-	-	N1
LEF1	-	-	N1
Gli2	-	+	SRR1
Stat3	+	+	SRR1
Oct4	+	+	SRR1
Brn1	-	+	SRR1

Transcription factor binding sites from [5] and their availability in JASPAR [21] or Transfac [22] (commercial version) as of 2010. "-" indicates that a position specific scoring matrix is **not **available, "+" indicates that it is. Instead of RARE, retinoic receptor RARA and RAR-gamma are available.

One should keep in mind that our hypotheses require experimental validation; some experiments will be reported in the next section. Following the hypothesis of "ubiquitous transcription" (see "Background"), we suggest that many of the following interpretations do have a small, but often negligible, probability to hold true in a specific biological context. Nevertheless, one or more interpretations may emerge as important new links in the transcriptional regulatory network, if more experiments are done. Patterns are ordered by branch length score [37]. The numbering of patterns in the UCSC tracks of some figures is not always consecutive; while patterns found only in species other than mouse are included in the ReXSpecies 2.0 pattern list for the respective regulatory region, they are not included in a figure using mouse as the reference genome.

#### Interpretation 1, the CR3 region (see also Figures 3 and 7 and Additional Files 2, 9, 10, and 17)

The CR3 region is of interest because there are no known binding sites (cf. Table 1).

The only module found by ReXSpecies 2.0 in the CR3 region (Figure 3) includes Smad (left) and Klf4/Stat (right), matching the module proposed in Tomlinson & Chambers (2009) [47], Figure six therein. The Smad3-Klf4 cooperation was also demonstrated by Hu et al, 2007 [48]. Moreover, we were able to demonstrate that both Smad and Klf4 binding have a regulatory impact on the CR3 region, see the next section.

Figure 7 displays further transcription factor binding sites gained by primates (green), as well as transcription factor binding sites gained by rodents (cyan). The specific sites for *primates *are Sp1, LRF, LFA1, MZF1, ZF5, ETF, HNF4, Churchill, MZF114 and Kid3; and the specific sites for *rodents *are FOXO1, CDPCR3, CIZ, FOXO3, FOXD1, Sox5, FOXL1, Kid3/ZNF354 and Prrx2. Since there are very few papers describing species-specific transcription of *Pou5f1 *or *Sox2*, or species-specific usage of transcription factors involved in pluripotency, currently there is no easy validation of these sites available.

#### Interpretation 2, the CR1 region (see also Figure 4 and Additional Files 3, 6, 11, 12, and 18)

Sorting the patterns found for CR1 by branch length score, the first six patterns with exception of the forth are describing "small modules". The binding sites they include are often also included in the "large modules" #4, #8, #9 and #12.

Pattern #1 (in Euteleostomi, but not human) is composed of Ets1 and ETF (a.k.a. TEF-4). Notably, it is long known that Ets1 activity is enhanced by TEF-4 (in COS-7 cells, derived from African green monkey, Chlorocebus [49]), leading to activity of *CTalpha. CTalpha *in turn regulates phosphatidylcholine biosynthesis, which is implied in mouse embryonic development [50], and its possible co-regulation with *Pou5f1 *may be reflected by this module.

Pattern #2 (in Euteleostomi and in Boreotheria) may relate to *Vitamin D receptor (Vdr) *expression, which plays a role in growth and differentiation. It consists of Wt1 and Zeb1 binding sites, both of which up-regulate the activity of the *Vdr *promoter (as does Sp1) [51]. Thus, *Vdr *activation may go hand in hand with Oct4/*Pou5f1 *repression by way of this module. Moreover, Zeb1 is an EMT inducer [52], and Wt1 is required for EMT in embryonic stem cells (see [53], their Figure four), providing another possible connection to repression of pluripotency and *Pou5f1*.

Pattern #3, ETF (Embryotrophic factor-3) and BRCA1 (Breast cancer type 1 susceptibility protein) in Euteleostomi (with few seemingly random exceptions) are both linked to proliferation, just by their names. However, investigating the literature and databases (Google Scholar, STRING) no other connection between these two putative stimulators of *Pou5f1 *could be found.

Pattern #4 features some of the earlier discussed transcription factors, as well further transcription factors that are known to be involved in pluripotency and differentiation (MyoD, Sp1, Zfx, Klf4, E2f1, Gata1). Notably, the Sp3 site (arrow, see also Table 1) matches a literature-curated site (of the wiki track), thus this is already experimentally validated.

Pattern #5 is found in all Boreotheria. The transcription factor ACAAT shows similarity to the binding motif of the Sox30 protein (5'-ACAAT-3' [54]). Unfortunately, no position specific scoring matrix for Sox30 is available in JASPAR nor in Transfac. Sox30 was proposed to play a role in differentiation of male germ cells [54]. GATA was discussed to play a potential role in germ cells [55]. It is possible that both differentiation-related transcription factors are involved in *Pou5f1 *regulation, suppressing its expression in the course of germ cell differentiation.

Pattern #6 in Boreotheria includes transcription factors that are implied in the development of the pancreas, CD44+ cells and photoreceptor cells. If it is functional, it may simply be inhibitory. This pattern also contains Pax and Gata3, which may work together in regulation of kidney development [56]. Pattern #8 is like Pattern #4 quite large. Even though it is predicted for the Boreotheria by parsimony, only parts of it are available for many of the Boreotheria including mouse and human. It contains many factors that are relevant for the control of pluripotency, such as COUP (matching the literature curated site, arrow, see also Table 1), Stat, Gata, and Esrrb, but no clear interpretation is possible.

Pattern #9 (Eutheria, by parsimony) overlaps with the literature-curated known binding site LRH-1 (arrow, see also Table 1). At the "far left" (in the homology-based figure), it includes Ets1 and TEF1; this part is not found in mouse (and not shown in the UCSC figure). Since it is not found in some other species either, it triggers a disharmonic TreeLogo (Additional files 11 and 12). LRH-1 may cooperate with some other transcription factors in the pattern, which are implicated into pluripotency, such as Klf4 and p300. Pattern #12 in Murinae yields an interesting hypothesis of the mouse- specific cooperation of the pluripotency-related transcription factor binding sites Zic2/3, Mycn, COUP-TF2 (which is experimentally validated, arrow, see also Table 1; COUP-TF2 also known as Arp1), and Yy1.

#### Interpretation 3, the N2/SRR1 region (see also Figure 5 and Additional Files 4, 7, 13, 14, and 19)

Part of the SRR1 region was already analyzed by ReXSpecies 2.0 in [5] (Figure seven therein). In that analysis, we zoomed into the small subsection of SRR1 that includes the Stat3 and Oct4/Brn1/2 binding sites experimentally validated in mouse: this subsection is conserved up to fish. We found patterns including the Stat3 and Oct4/Brn1/2 binding sites, and two further patterns that combine predicted binding sites of transcription factors of dual (neural as well as pluripotency) relevance, possibly reflecting the known dual role of the N2/SRR1 region [5]. In Figure 5, we show the patterns that we observe if we zoom out and analyze the entire SRR1 region. In particular, pattern #3 includes matches to the experimentally validated Oct4/Brn1/2 binding sites, designated Brn2 and OCTx (arrows, see also Table 1). In the earlier zoom-in analysis, the first Oct4/Brn1/2 binding site was out-of-scope and could not be found; this first site is now found, in pattern #3, together with the second validated Oct4/Brn1/2 binding site, and it co-localizes with Sox-related binding (Sry) as well as Stat-related binding (Stat5A/6). Pattern #3 includes, besides others, Nobox and Pdx1 binding sites on the left that are missing in mouse (and not shown in the figure). Therefore, not the entire pattern is found in mouse/rat. However, it is estimated by parsimony to be gained first in Tetrapods.

The first two tracks (patterns #1 and #2) are surprising; PU.1 and Gata1 are known as antagonistic regulators of hematopoiesis [57] and no connection with pluripotency or (early) neural development has been described so far. Thus, either 1. the patterns are false positives, or they contribute to ubiquitous transcription (which is the most likely scenario; see "Background"); or 2. PU.1 and Gata1 are involved in pluripotency or early neural development, that is they are "moonlighting" in addition to their well-known role; or 3. the region under investigation is involved in hematopoiesis (implying with high probability that *Sox2 *is); or 4. PU.1 and Gata1 could fulfill a repressive function on the Sox2 locus, to ensure that there is no ectodermal pattern being expressed in the hematopoietic cells.

There is a hint of evidence for scenario (2), since the *Sox2 *N2 region regulates the anterior neural plate [45] and Rekhtman et al [57] report that after injection of PU.1 RNA, Xenopus "embryos also displayed defects unrelated to hematopoiesis in the developing dorsal axis, seen first as an uneven neural plate (not shown) and resulting in abnormally shaped tails." It should be noted that although the PU.1 pattern (pattern #1) is inferred to be amniotic (same as the GATA pattern, pattern #2), it also appears in some fish (Percomorpha), where it must have been gained independently, according to parsimony. Then again, it is more likely that it was gained once in Euteleostomi, but lost in zebrafish and frog.

In the next two tracks, patterns #4 and #6 are displayed. They both feature Stat1, which is slightly upstream of the validated Stat3 binding site. They also feature MOVO-B/Ovol2, which is required for embryonic development, in particular of the cranial neural tube [58]. Finally, the last two patterns occur in very few species, reflected by their low branch length score. If they are not false positive, they are likely a "negligible" part of ubiquitous transcription.

#### Interpretation 4, the N1 region (see also Figure 6 and Additional Files 5, 8, 15, 16, and 20)

The N1 region regulates the anterior neural plate [45]; it features few binding sites that were experimentally validated in mouse (a tandem Lef1 binding site, and a single Fgf binding site [45]). However, Transfac-based computational analyses cannot find Fgf binding sites, because there is no model (see Table 1). As reported by [45] (their Figure six), the first Lef1 binding site is found in all tetrapods that were investigated, whereas the second Lef1 binding site was lost (or never gained) in Xenopus frog. In line with this observation, we infer that patterns with the second site were gained in amniotes (patterns #7, #10, #17, red arrows), whereas patterns with the first site are inferred to be gained in tetrapods (patterns #1 and #14, blue arrows). All these patterns include a variety of other predicted binding sites, with no clear overrepresentation of specific groups of transcription factors. Apart from the group of patterns that include Lef1, a second group of patterns is worth to be discussed (#2, #6, #8 and #12). These patterns include Oct and/or Sox motifs (green arrows). In particular, the Oct/Sox (Sry/Sox5/Oct1) motif of pattern #2 may indicate an involvement of N1 not just in neural development, but also in pluripotency.

### Experimental validation

**Figure 8:**
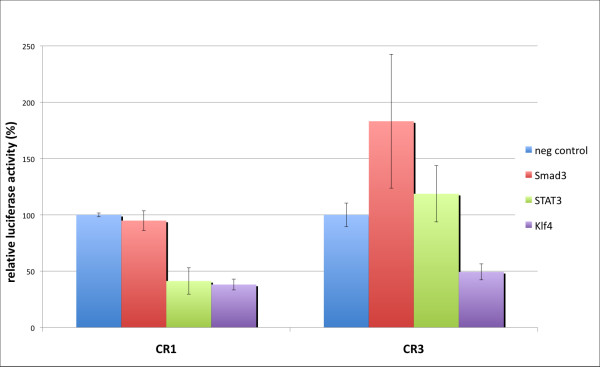
**Luciferase assay of the CR1 and CR3 regions**. Transactivation activity of Smad3, Stat3 and Klf4 on the conserved regions (CR1 and CR3) was investigated by the dual-glow luciferase assay. Smad3 shows elevated transactivation activity on CR3. Stat3 leads to transcriptional repression on CR1. Klf4 binding results in a repressive effect on CR1 and CR3.

In order to evaluate the benefits of ReXSpecies 2, transactivation of three transcription factors to the conserved regions of the *Pou5f1 *regulatory region [44] was investigated by luciferase assays (see Figure 8). LIF (Leukemia inhibitory factor) was used to trigger the Stat-pathway and resulted in a significant transcriptional decrease in CR1, while it showed no effect on CR3 in comparison to the negative control. The straightforward interpretation is that only the CR1 region has a functional Stat binding site in the context of our experimental setup. This functional binding site may match the one predicted to be part of pattern #8. Activin A was used to activate the Smad-pathway, facilitating Smad3 binding to its downstream target genes. The activation of Smad3 showed a modest but reproducible increase of the luciferase activity on CR3 while it shows no regulatory effect on the CR1. This matches the ReXSpecies 2-based results for these two conserved regions that we presented above; no Smad binding is predicted for the CR1 region, in contrast to the CR3 region. Klf4 overexpression led to a transcriptional repression in the conserved regions 1 and 3 in our assay; Klf4 was predicted to bind to both conserved regions.

Since it was shown that Klf4 partners with the histone acetyltransferase p300, and the luciferase system lacks the epigenetic landscape of the endogenous regulatory elements, the transcriptional repression may not represent the full picture of Klf4 function on the regulatory region of the *Pou5f1 *gene. However, the observed effects on the transactivation activity clearly indicate that Stat binds to the CR1, Smad3 binds to the CR3, and Klf4 binds to the CR1 and CR3 regions. These experimental data validate in part the predicted modules and highlight the value of the ReXSpecies 2 filters for experimental researchers.

## Conclusions

### Exploration of regulatory sequences made easier

The examples show that there are usually large amounts of filtered data returned by ReXSpecies 2.0, which must still be vetted by an expert. This is not necessarily the fault of ReXSpecies, but it is due to the fact that only sequence information, but no gene expression data, etc., which is not usually available, is used as input. Moreover, as discussed in the next section, the large amount of filtered data returned by ReXSpecies 2.0 may reflect reality at least to some degree. Nevertheless, we believe that scientists benefit from its first-line filtering, and they moreover can benefit from our visualization and from the interactive tools we provide. The incorporation of evolutionary considerations can help identify true positive transcription factor binding site predictions. The *putative evolution *of (predicted) transcription factor binding sites is thus displayed, going beyond common visualization approaches such as genome browsers. ReXSpecies 2.0 helps to explore the large amount of predictions returned by matching classic position specific scoring matrices. It automates many steps from downloading the data to running position specific scoring matrix software, and it aligns sequences and predictions in order to find conserved patterns in the output data. It then converts the results into useful formats (PDF, PS, SVG, WMF, XLS,...).

Furthermore, it interfaces with genome browsers to analyze the conserved patterns in other contexts. Thus, integrative and comparative studies with known tracks at UCSC and other genome browsers are made easy and supplementing results of such regulation studies with other published work is facilitated. Scientists can therefore find relevant transcription factor binding site predictions for their work.

### Ubiquitous transcription and the interpretation of ReXSpecies results

To exemplify the advantages of evolution-aware analyses of transcriptional regulation using ReXSpecies 2.0, we have derived some plausible hypotheses for murine Oct4/*Pou5f1 *and *Sox2 *regulation. We verified some of these experimentally. Even for the other predictions, many of which do not match known transcription factor binding sites, reasonable interpretations are often possible. This is in line with the hypothesis of ubiquitous transcription (that is, transcription may be more common than previously thought, see the Background section), because one explanation for ubiquitous transcription is the possibility that transcription factor binding may also be more common than previously thought. As discussed in the Background section, ubiquitous transcription sheds new light onto computational predictions, *hypothesizing *that most of them are in fact real, as are the modules we find, even though in many biological contexts (of transcription factor abundance and/or DNA accessibility) they are utilized only to a negligible degree.

## Implementation

### Prediction of transcription factor binding sites

#### Sequence based prediction of transcription factor binding sites

Usually, in-silico transcription factor binding site predictions are based on the DNA base sequence of regulatory sequences. Therefore, a table (position specific scoring matrix) of log-likelihoods (or log-odds) is used to detect transcription factor binding sites in a regulatory sequence; each column in that table stands for a position in the binding sequence, and each row represents a base (A, T, C, or G) [59]. The entries in the table express how often a base occurs at a position in a set of known binding sites of the transcription factor that the position specific scoring matrix belongs to.

ReXSpecies 2.0 uses PoSSuM [10] in combination with the freely available JASPAR database [21] for the purpose of searching for transcription factor binding sites. More transcription factor binding site models besides JASPAR may be uploaded in Transfac file format [22,60]. For our examples, we used the 2010 Transfac position specific scoring matrix library (commercial version), [22]. Table 1 features a detailed list of all transcription factor binding sites curated in [5] and found in the regions investigated here, indicating for which a position specific scoring matrix exists in our position specific scoring matrix libraries from Jaspar and/or Transfac [21,22].

#### Alignment and clustering of transcription factor binding site predictions

ReXSpecies 2.0 fetches the regulatory sequence under investigation specified by absolute genome coordinates or by coordinates relative to the transcription start site of a gene from the UCSC genome browser [9]. At UCSC, the multiz alignment tracks [9] provide homologous sequences of other species. Alternatively, users may upload homologous sequences of their own. The multiz-alignment was pre-calculated by UCSC for 30 different species in the mouse genome and 17 in the human genome. This alignment is used by default as the sequence alignment. If activated, ReXSpecies 2.0 can employ several sequence alignment tools to redo the alignment, per default muscle [61] is used. Then, ReXSpecies aligns the transcription factor binding site predictions such that their sequence coordinates are converted to alignment coordinates considering gaps, which have been introduced by the alignment tool, as described in [7]. Afterwards, ReXSpecies tries to identify homologous transcription factor binding sites. Two binding sites are considered to be homologous if they essentially overlap and if they are bound by homologous transcription factors based on HomoloGene [42]. "Essentially overlapping" means that the beginning or the ending of the two aligned predictions overlap. Beginning and ending are two regions in the alignment related to a transcription factor binding site prediction. If the sequence length (not counting gaps) of the prediction in base pairs is called *l*, its start position in the alignment in alignment coordinates (i.e. including gaps) is called *s *and its end position in the alignment (in alignment coordinates) is called *e*, then the *beginning *is the region in the alignment from *s *to *s *+ *l *(in alignment coordinates), and the *ending *is the region from *e - l *to *e*. These calculations are done to prevent predictions with binding sites with larger gaps, i.e., disrupted binding sites containing insertions gained during evolution from being considered homologous to all binding sites for similar factors within that insertion. Such larger gaps could also be the result of alignment errors. Each prediction is then assigned a branch length score as proposed in [37]. Predictions can be filtered based on different scores, e. g. the matrix similarity score [23], the branch length score [37], or the E-value. Additionally, filtering based on other features, such as target species/clade of the model or name of the transcription factor is possible.

Finally, considering each species separately, redundant predictions are identified. Predictions are *clustered *if they share the start, middle $\left(middle=\left\lfloor \frac{start+end}{2}\right\rfloor \right)$ or end (alignment-)coordinate, their length in base pairs is the same and at least one of the following conditions is true:

• The names of the transcription factor binding sites predicted are similar (not considering upper/lower case, spaces, numbers).

• The predictions are made for homologous transcription factors (based on HomoloGene [42]).

**Figure 9:**
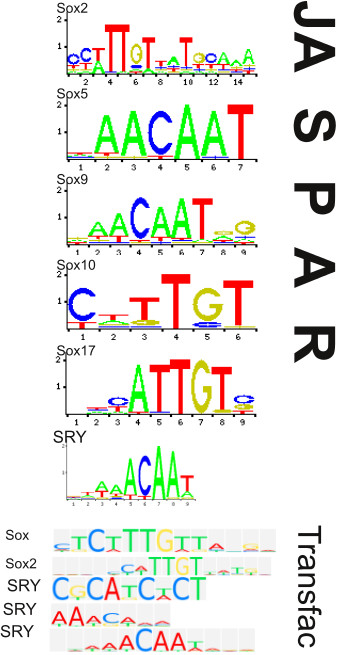
**Different Sox transcription factor binding site motifs**. The sequence logos [41] for different Sox paralogs from different sources (JASPAR and Transfac [21,22]) are shown. Transfac uses a slightly different color coding (colors of A and T are switched). Obviously, Sox5 and Sox9 match nearly the same motifs, Sry matches a closely related one. The reverse complement of the ACAA core of these motifs is furthermore part of the other JASPAR motifs (TTGT in Sox2, Sox10 and Sox17). TTGT is also part of the first two Transfac motifs shown and ACAA is part of two of the three Transfac Sry motifs. The Transfac Sox-Motif is a family motif that is assigned to many different Sox-paralogs (Sox2, Sox3, Sox4, Sox5, L-Sox5, Sox6, Sox6 isoforms, Sox8, Sox9, Sox10, Sox11, Sox12, Sox13, Sox14, Sox15, Sox18, Sox20, Sox21, SRY, SoxLZ, Sox-xbb1) from different taxa (Mouse, Rat, Chicken, Human, the whole Mammalia taxon, and some more less known model species).

Clustering overlapping transcription factor binding site predictions of similar name can be done, because a similar name in the Jaspar database [21] implies that the transcription factors in question are homologs. Clustering has the effect that many probably equivalent overlapping predictions are replaced by only one prediction. Therefore, fewer matches are reported. For example, Figure 9 displays the binding site motifs of various transcription factors of similar name (Sox). These are all Sox homologs. If the overlap conditions are met, they are clustered, yielding only one prediction for all overlapping Sox matches named "Sox". In particular, ReXSpecies identifes redundancy by removing numbers from the transcription factor names (Sox2 → sox, Sox17 → sox) and comparing the resulting strings (sox). The rigid matching rule (same start, middle or end coordinate plus same length), however, prevents the clustering of predictions that are not redundant. On the other hand, the user can completely turn off clustering. In two cases predictions may also be *joined *if they simply overlap (relaxed matching rule): a) if they are made for the same binding transcription factor, or b) if they are at least made for a homologous transcription factor based on HomoloGene [42]. Note that the clustering of redundant predictions, which is done right after detection, is distinct from the joining of homologous predictions. Joined and clustered predictions are assigned a combined name e. g., Sox for the Sox2/Sox17 predictions. Likewise, synonyms such as *Pou5f1 *and Oct4 are assigned a name such as Pou5f1 Oct4. If needed, manual joining is also possible.

#### Phylogenetic tree with labelling by inferred ancestral transcription factor binding site predictions

After the generation of position specific scoring matrix-based binding site predictions for the sequences, ReXSpecies (since 1.0) labels a phylogenetic species tree [27-29] (Figure 2) using the binding site predictions as labels for the leaves of the tree and employing the Fitch parsimony [30] method to reconstruct the labels for the ancestral species in the tree. Thus, the extant species are labelled with the binding site predictions and the inner nodes are inferred by parsimony. Alternatively, the common tree originating from either the NCBI taxonomy browser [62], the tree used for the multiz alignment [28], or a user-provided tree can be labelled.

The parsimony method minimizes the number of changes that have to be assumed to explain the data given for the extant species in a phylogenetic tree. There are also statistical methods (e.g. Bayesian statistics [63] and maximum likelihood methods [64]) to predict ancestral states, but they do not always predict fewer false positives [65]. Since it is quite fast and generates results even if data is noisy and sparse, we decided to use the parsimony method. Nevertheless, parsimony-generated models of binding site evolution tend to overfit so that ReXSpecies also suffers from that overfitting. Interpretations of ReXSpecies output must take care of this fact. In particular in combination with the noisy underlying alignment data this causes erroneous predictions of losses, if parts of the alignment are wrong or missing.

#### Finding groups of transcription factor binding sites gained/lost together

Once the tree has been labelled, ReXSpecies 2.0 finds gain/loss patterns. As defined in the background section, a *transcriptional module *is defined as *a set of transcription factor binding sites, and a gain/loss pattern is defined as a transcriptional module, for which we find a gain or loss in evolution*. In other words, ReXSpecies 2.0 finds sets of two or more transcription factor binding sites that are separated by a specific number of base pairs, all of which are either gained or lost at the same node of the phylogenetic tree; the transcription factor binding sites making up a module must have the same conserved distance (in base pairs) in all species where they are present. See also Additional File 1, Part I for the pattern detection algorithm. By default, we focus on transcription factor binding site gains only, because we consider gains more informative than losses. The patterns are then ranked using a branch length score as proposed in [37]. Furthermore, by default we ignore gains and losses in the extant species, because of the large amount of binding site matches in these. There are far fewer matches to consider if we base our analysis on parsimony reconstruction of ancestral labels in the species tree, because parsimony-based reconstructions for an inner node are based on two or more predictions for the leaves (the extant species). As described in the last section, these reconstructions may nevertheless be noisy.

As an alternative to gain/loss patterns, ReXSpecies 2.0 supports the calculation of branch length scores as proposed by [37] for each transcription factor binding site prediction (as well as for gain/loss patterns), and to use these scores for ranking and filtering transcription factor binding site predictions.

### Experimental validation

The dual-glow luciferase assay (Promega) was used to monitor the binding of transcription factors to the regulatory elements of the Pou5f1 gene. The conserved regions CR1 and CR3 were cloned into the pGL3 vector, driving the expression of firefly luciferase. 1 ug pGL3 was cotransfected with 10 ng of pRL (encoding renilla luciferase) for normalization. 20 × 10000 Human embryonic kidney cells (293T) were cultured in DMEM (1000 mg/l glucose) supplemented with 10% FCS, non-essential amino-acids and Glutamine (2 mM). LIF (2000 u/ml) and Activin A (100 ng/ml) were used to trigger STAT3 and Smad3 pathways respectively. Klf4 was cloned into pMX retroviruses and co-transfected into 293T cells together with reporter and effector constructs using Fugene (Roche).

## Future work

We plan to integrate some more genomes from UCSC [6]. Furthermore, we plan to implement a software module that finds homologous promoter sequences by running BLAST searches in genome data or using the HomoloGene database [42], or based on alignment-free promoter search [15]. We would like to be able to predict gene transcriptional regulation networks that could be analyzed using tools such as Cytoscape [66] and ExprEssence [67], and that could be viewed using Cytoscape or VANLO [68].

## Availability and requirements

Project name: rexspecies

Project home page: http://sourceforge.net/projects/rexspecies

Operating system: Web application running on Linux [8]

Programming language: Perl

Other requirements: bioperl, muscle, mysql, ldap, MrBayes, PoSSuM

License: GNU LGPL [69]

## Authors' contributions

SS wrote the software, did the ReXSpecies part of the analyses, generated all figures and wrote large parts of the text. GF wrote the interpretations of the ReXSpecies output and did further literature studies; furthermore, he supervised the project. Experimental validation of some of our results was performed and described by DE under the guidance of HS. All authors read and approved the final manuscript.

## Supplementary Material

Additional file 1 **Supplementary methods and results**. The pattern search algorithm is described. Furthermore, all additional output files that are not described in the main article are listed here. Click here for file

Additional file 2 **BED detail format file with the annotations for the CR3 region**. This file can be uploaded to UCSC. It contains the transcription factor binding site pattern found in the conserved murine CR3 region near the *Pou5f1 *gene. All transcription factor binding sites are annotated with their STRING [31,32] and their iHOP [33,34] annotations, if available. Click here for file

Additional file 3 **BED detail format file with the annotations for the CR1 region**. This file can be uploaded to UCSC. It contains all transcription factor binding site patterns found in the conserved murine CR1 region near the *Pou5f1 *gene. All transcription factor binding sites are annotated with their STRING [31,32] and their iHOP [33,34] annotations, if available. Click here for file

Additional file 4 **BED detail format file with the annotations for the SRR1 region**. This file can be uploaded to UCSC. It contains all transcription factor binding site patterns found in the conserved murine SRR1 region near the *Sox2 *gene. All transcription factor binding sites are annotated with their STRING [31,32] and their iHOP [33,34] annotations, if available. Click here for file

Additional file 5 **BED detail format file with the annotations for the N1 region**. This file can be uploaded to UCSC. It contains all transcription factor binding site patterns found in the conserved murine N1 region near the *Sox2 *gene. All transcription factor binding sites are annotated with their STRING [31,32] and their iHOP [33,34] annotations, if available. Click here for file

Additional file 6 **CR1 patterns, Homology-based figure**. The patterns and the transcription factor binding sites found in the murine CR1 region. See Figure 7 for further explanations. Click here for file

Additional file 7 **SRR1 patterns, Homology-based figure**. The patterns and the transcription factor binding sites found in the murine SRR1 region. See Figure 7 for further explanations. Click here for file

Additional file 8 **N1 patterns, Homology-based figure**. The patterns and the transcription factor binding sites found in the murine N1 region. See Figure 7 for further explanations. Click here for file

Additional file 9 **CR3 pattern, table format**. The pattern found in the murine CR3 region. See also Additional File 3 for a more detailed version of this table. Click here for file

Additional file 10 **CR3 pattern, detailed table format**. The pattern found in the murine CR3 region, detailed version of Additional File 2. Click here for file

Additional file 11 **CR1 patterns, table format**. The patterns found in the murine CR1 region. See also Additional File 7 for a more detailed version of this table. Click here for file

Additional file 12 **CR1 patterns, detailed table format**. The patterns found in the murine CR1 region, detailed version of Additional File 6. Click here for file

Additional file 13 **SRR1 patterns, table format**. The patterns found in the murine SRR1 region. See also Additional File 11 for a more detailed version of this table. Click here for file

Additional file 14 **SRR1 patterns, detailed table format**. The patterns found in the murine SRR1 region, detailed version of Additional File 10. Click here for file

Additional file 15 **N1 patterns, table format**. The patterns found in the murine N1 region. See also Additional File 15 for a more detailed version of this table. Click here for file

Additional file 16 **N1 patterns, detailed table format**. The patterns found in the murine N1 region, detailed version of Additional File 14. Click here for file

Additional file 17 **CR3 pattern, Alignment based figure**. The pattern and the transcription factor binding sites found in the murine CR3 region. In principle, the elements of this figure correspond to those in Figure 7. Obviously, the alignment-based figures are much larger than their homology-based version. In contrast to the homology-based figure type, the leaves of the species tree on the left are corresponding to the sequences of the extant species on the right. The sequences are shown in a sequence alignment and the transcription factor binding site predictions are plotted below the sequences. Because the species belonging to each prediction is obviously determined by its vertical position (the leaves are not color coded here), only the thicker part of the colored overlays (see Figure 7) is informative and thus it is shown; it denotes the pattern by referring to its color. To keep the figures readable, by default only the first 10 patterns (ordered by branch length score) are shown in the alignment-based figures. Click here for file

Additional file 18 **CR1 patterns, Alignment based figure**. The patterns and the transcription factor binding sites found in the murine CR1 region. See Additional File 17 for an explanation. Click here for file

Additional file 19 **SRR1 patterns, Alignment based figure**. The patterns and the transcription factor binding sites found in the murine SRR1 region. See Additional File 17 for an explanation. Click here for file

Additional file 20 **N1 patterns, Alignment based figure**. The patterns and the transcription factor binding sites found in the murine N1 region. See Additional File 17 for an explanation. Click here for file
