# Supplementary material for: Visualization and Exploration of Conserved Regulatory Modules Using ReXSpecies 2
Source: BMC Evol Biol. 2011 Sep 24;11:267. doi: 10.1186/1471-2148-11-267 (PMC3203875; doi:10.1186/1471-2148-11-267)
Supplement: Additional file 1 — Supplementary methods and results. The pattern search algorithm is described. Furthermore, all additional output files that are not described in the main article are listed here. [file 1471-2148-11-267-S1.PDF]

Part I

Algorithm that finds gain/loss patterns

1 Purpose

The following algorithm was designed to find all sets of gains and/or losses of such transcription factor binding sites that occur at least along two edges in the species tree. Please see Figures 1 and 2. Figure 1 shows some of the patterns, we consider to be interesting. A, B, C, and D are sequences of different species, the species can be extant or ancestral. If we do not have the exact sequences of the ancestral species, Fitch parsimony can be used in order to reconstruct transcription factor binding sites for these ancestral species. Patterns that we would consider to be interesting are highlighted in green, orange, and magenta. Below the image, the sets of species are shown, where the patterns occur at. 1, 2, and 3 are different states (presence or absence) in the transcription factor binding sites that occur along the evolutionary edge, leading to the corresponding species node: E.g. 1 may be the presence of a Sox2 site, 2 may be the absence of a Nanog site, and so on. The number expresses the binding transcription factor and if it is present or absent.

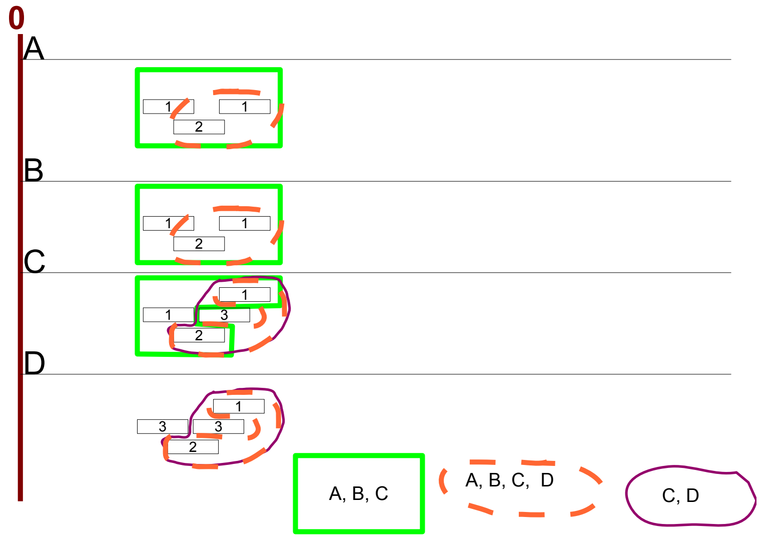

Figure 1: "Interesting" *absolute* patterns in the alignment
The letters A – D stand for different species. The numbers 1 – 3 represent binding sites of different transcription factors 1 – 3 (or, generalized, presence of absence of binding sites of transcription factors 1 – 3). For factor (state) 1, in particular, two binding sites exist in species A – C.

First, we concentrate on finding patterns of transcription factor binding site gains/losses, that occur at exactly the same alignment positions. This is shown in Figure 1.

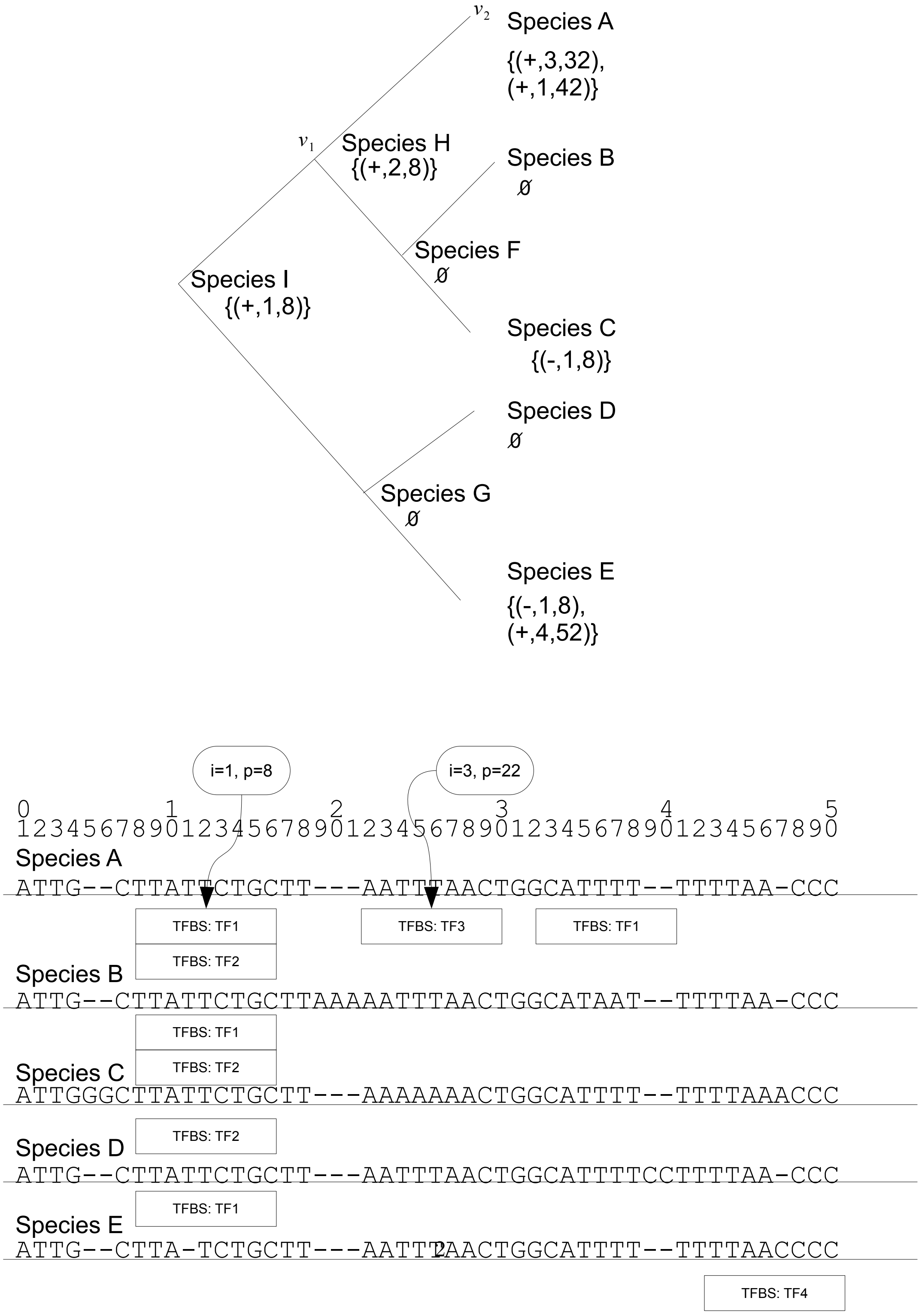

Figure 2: Interesting patterns in alignment and tree, definitions visualized

2 Definitions

Besides their verbal definition, the following terms are visualized in Figure 2 also.

- The *species tree* $T = (V, E), E \subseteq \{(v_1, v_2)|v_1, v_2 \in V\}$ is a rooted tree (with root $r \in V$). The elements of V are called vertices, each representing species. All edges in E are directed, so that for each edge (v_1, v_2) the first vertex v_1 is the one that is closer to the root.
- An *event* is the gain or loss of a transcription factor binding site at a certain position in the alignment along the lineage from one species to another. It is denoted as a triple $(s, i, p), s \in \{-1, +1\}; i, p \in \mathbb{N}$, where $(s = +1)$ means gain and $(s = -1)$ means loss of the transcription factor i at position p in the alignment. Then, $\Omega = \{(s, i, p)|s \in \{-1, +1\}; i, p \in \mathbb{N}\}$ is the set of **all** possible events.
- Based on the parsimony annotation of the edges, the *pattern* $\Psi(v)$ of a vertex $v \in V$ is a subset of Ω. It is the set of **all** gains and losses of transcription factor binding sites along the edge $(*, v)$ pointing to the species at vertex v.
- In the following, the *family* F_m contains all intersections generated until step $m \in \mathbb{N}_0$ of the algorithm.

3 Algorithm

The following algorithm find_common_patterns finds all common patterns in the species tree T for all subsets of vertices $U \subset V$

INPUT: $\{\Psi(v), \forall v \in V\}$

1. $F_0 := \{\Psi(v)|v \in V\}; m := 0$

   DO {

2. $F_{tmp} := F_m;$

3. $F_{m+1} := F_m \cup \{A \cap B|A \neq B \wedge A, B \in F_m\};\ m := m + 1$

   }

   WHILE $F_{tmp} \neq F_m$

OUTPUT: $F := F_m$

We will write that a pattern is found by find_common_patterns, if it is included in F.

In step 3, find_common_patterns adds all sets $A \cap B$, with $A \neq B$ and $A, B \in F_m$, i.e. it adds all intersections of each two sets in the current family F_m. The algorithm stops as soon as no new set is generated by step 3, so the closure with respect to intersection is returned, i.e. all intersections of each two sets in F are also in F.

4 Relative patterns

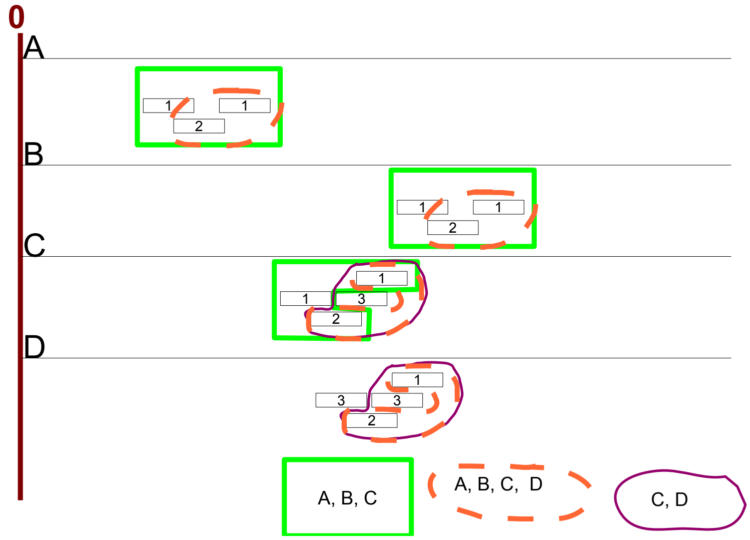

Figure 3: "Interesting" *relative* patterns in the alignment
The letters A – D stand for different species. The numbers 1 – 3 represent binding sites of different transcription factors 1 – 3 (or, generalized, presence of absence of binding sites of transcription factors 1 – 3). For factor (state) 1, in particular, two binding sites exist in species A – C.

The algorithm above can be generalized to find all sets of gains/losses of transcription factor binding sites (events) along two or more edges in the species tree, but with relative positions as shown in Figure 3. *Relative* means, that in a set of events (pattern) considered to be in common, the relative distances between each pair of events in the pattern are the same for each occurrence in the species tree, but the absolute start positions of the pattern in the alignment may differ from species to species (or even within one species, if the same pattern is gained/lost twice), e.g. a pattern would be the gain of a Sox2 site followed by the gain of an Oct4 site 8 base pairs downstream from that Sox2 site, and a loss of a c-Myc site 16 base pair downstream from the Sox2 site then. (An *absolute* pattern as searched by the find_common_patterns algorithm would be the gain of a Sox2 site at position 209 combined with the gain of an Oct4 site at position 217, and the loss of a c-Myc site at position 225)

In order to do that generalization, a pre-processing step and some post-processing was introduced. For each element F_0^i of $F_0 = \{F_0^1, F_0^2, \ldots, F_0^{|F_0|}\}$ all suffixes are generated and *added* to F_0, i.e. for each alignment position $p \in \mathbb{N}$ all subsets of F_0^i that contain all transcription factor binding sites of F_0^i with a position right from p are added to F_0, including the initial elements F_0^i. Running find_common_patterns on this modified input then finds such relative sets. Finally, it has to remove certain redundancies introduced by the pre-processing.

4.1 Definitions

First we need some definitions. The function leftmost assigns to each pattern Γ the set of its elements with minimum position in the alignment:

$$\begin{aligned} \text{leftmost} : \mathcal{P}(\Omega) \rightarrow \mathcal{P}(\Omega) \\ \text{leftmost}(\Gamma) \mapsto \{(s,i,p') \in \Gamma \mid p' = \min_{(s,i,p)\in\Gamma} p\} \end{aligned} \tag{1}$$

Next, we define a shift operation denoted by superscript shift distances:

$$n \in \mathbb{Z} \setminus \{0\}, \Gamma \in \mathcal{P}(\Omega) : \Gamma^{(-n)} = \{(s,i,p-n) \mid (s,i,p) \in \Gamma\} \tag{2}$$

Another shift operation needed is a shift to the zero position:

$$\Gamma \in \mathcal{P}(\Omega) : \Gamma^{(0)} := \Gamma^{(-n)} \text{ for } n = \min_{(s,i,p)\in\Gamma} p. \tag{3}$$

The set of vertices, for which a given common pattern is part of their annotation (pattern) is the image of the function ν:

$$\begin{aligned} \nu : \mathcal{P}(\Omega) \rightarrow V \\ \nu(\Gamma) \mapsto \{v \in V | \Gamma \subseteq \Psi(v)\}. \end{aligned} \tag{4}$$

4.2 Algorithm find_relative_common_patterns

1. $F := \{\Psi(v)^{(0)} | v \in V\}$ /* all patterns, shifted to position 0 */

   DO {

2. $f := |F|$

3. $F := F \cup \{(\Gamma \setminus \text{leftmost}(\Gamma))^{(0)} \mid \Gamma \in F\}$

4. } /* adding subsets, removing the first (left most) element, shifted to position 0 */

   WHILE ($f \neq |F|$) /* While new sets were generated by step #3 */

5. Run modified version of find_common_patterns

   (a) $F_0 := F;\ m := 0$ /* Different input */
   DO {
   (b) $F_{tmp} := F_m; m := m + 1$
   (c) $F_m := F_{m-1} \cup \{A \cap B | A \neq B \wedge A, B \in F_{m-1}\}$
   (d) }
   WHILE $F_{tmp} \neq F_m$ /* While new sets were generated by step #6c */

6. $F_{result} := F_m$

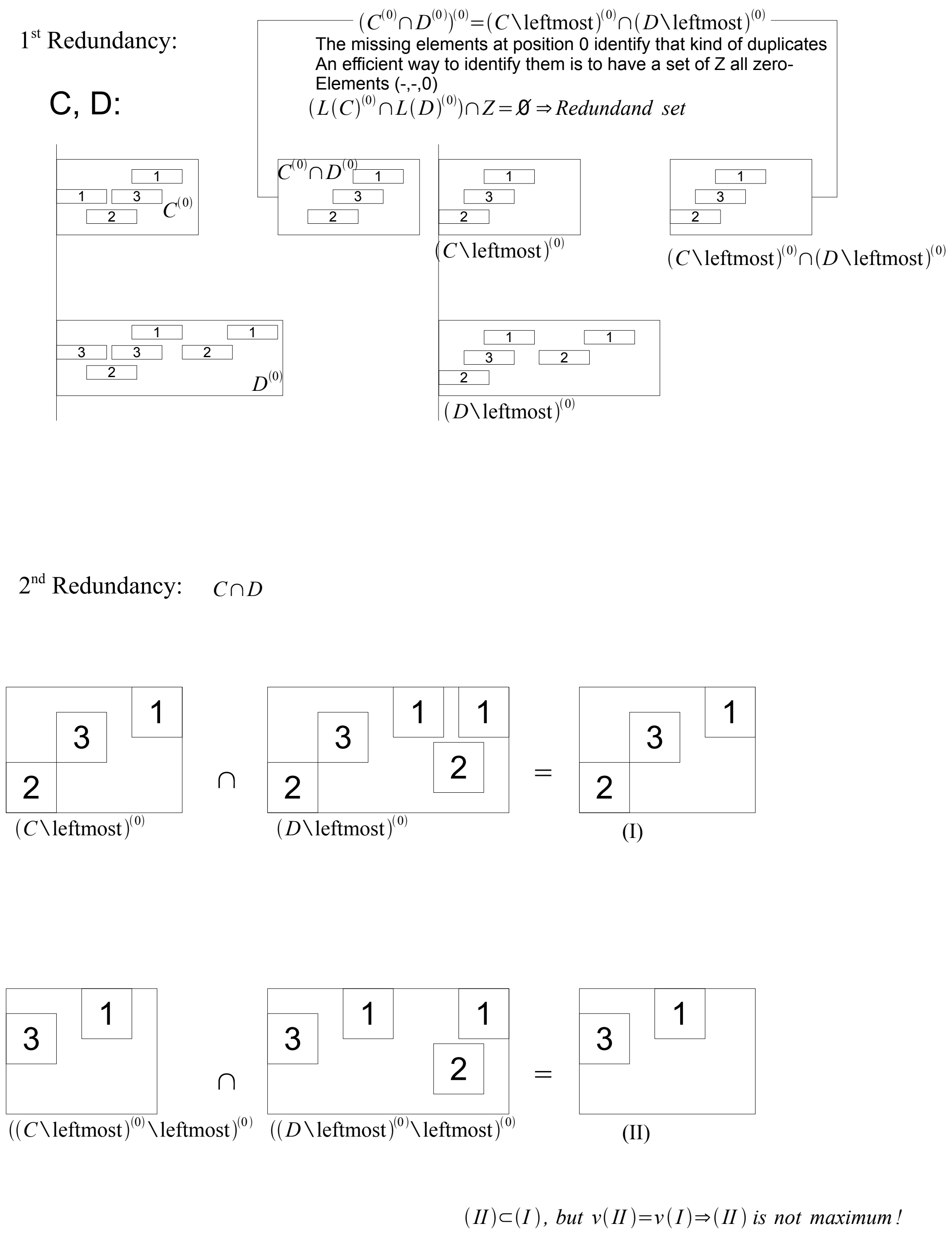

Figure 4: Two kinds of redundancy

In this algorithm two kinds of redundancy exist (see Figure 4). The first one (1st Redundancy in Figure 4) hides in the intersection step: If two (or more) patterns L_i have suffixes with common non-empty intersections but their prefixes differ, the resulting sets will be added twice: Once by the pre-processing as suffixes of those L_i and once by the intersection step, as intersections of these L_i, that will remove the different prefixes too. In the latter, the algorithm would then move the pattern to zero, so that duplicates were gained.

Another sort of redundancy results from the modified input (2nd Redundancy in Figure 4): Because of the new input patterns generated as suffixes of patterns, we get all suffixes of patterns, even if they are not maximum (have not maximum number of events) for any set of vertices. This is addressed by a new post-processing step (see below). Changes are marked using an asterisk (*).

1. $F := \{\Psi(v)^{(0)} | v \in V\}$ /* all patterns, shifted to position 0 */

   DO {

2. $f := |F|$

3. $F := F \cup \{(\Gamma \setminus \text{leftmost}(\Gamma))^{(0)} \,|\, \Gamma \in F\}$

4. } /* adding subsets, removing the first (left most) element, shifted to position 0 */

   WHILE ($f \neq |F|$) /* While new sets were generated by step #3 */

5. Run modified version of find_common_patterns

   (a) $F_0 := F;\ m := 0$ /* Different input */

   DO {

   (b) $F_{tmp} := F_m; m := m + 1$

   (c) * $F_m := F_{m-1} \cup \{(A \cap B)^{(0)} | A \neq B \wedge A, B \in F_{m-1}\}$

   (d) }

   WHILE $F_{tmp} \neq F_m$ /* While new sets were generated by step #6c */

6. * $F_{intersected} := F_m$

   /* Post-processing: Remove all patterns that are sub-patterns of a pattern that is denoted at exactly the same vertices */

   (a) $F := F_m \setminus \{\Gamma_1 \in F | \exists \Gamma_2 \in F, z \in \mathbb{Z} : \Gamma_1 \subset \Gamma_2^{(z)} \wedge \nu(\Gamma_1) = \nu(\Gamma_2)\}$

7. $F_{result} := F$

A more efficient way to handle the first kind of redundancy is not to add the redundant sets generated by the intersection step, because these redundant sets are not zero shifted. This can be detected easily, because the intersection of the redundant sets with the set of all zero-positioned elements is empty. This set of zero-positioned elements is initially generated to render the algorithm more efficient. This detection works faster than moving a subset to zero, because the sets are coded as a bit vector internally (a list of zeros and ones; a one at the n-th position in that vector means, that the n-th element is part of the coded set). So, intersection of two sets is simply done by the

logical AND operation while moving a pattern to zero needs to decode a bit vector, to shift all positions and to re-encode that vector again.

The algorithm with that additional modification is shown below. Changes are marked using an asterisk (*).

1. $F := \{\Psi(v)^{(0)} | v \in V\}$ /* all patterns, shifted to position 0 */
   * $Z \subseteq \Omega, Z := \{o^{(0)} | o \in \Omega\}$ /* to identify elements, that are at position 0 using a bitwise and operation */

   DO {

2. $f := |F|$

3. $F := F \cup \{(\Gamma \setminus \text{leftmost}(\Gamma))^{(0)} \mid \Gamma \in F\}$

4. } /* adding subsets, removing the first (left most) element, shifted to position 0 */

   WHILE ($f \neq |F|$) /* While new sets were generated by step #3 */

5. Run modified version of find_common_patterns

   (a) $F_0 := F;\ m := 0$ /* Different input */
       DO {
   (b) $F_{tmp} := F_m; m := m + 1$
   (c) * $F_m := F_{m-1} \cup \{A \cap B | A \neq B \wedge A, B \in F_{m-1} \wedge (A \cap B \cap Z \neq \emptyset \Leftrightarrow A \cap B = (A \cap B)^{(0)})\}$
       /* Only add intersections of patterns from F_{m-1}, that do contain a $(\cdot, \cdot, 0)$ */
   (d) }
       WHILE $F_{tmp} \neq F_m$ /* While new sets were generated by step #6c */

6. $F_{intersected} := F_m$ /* Post-processing: Remove all patterns that are sub-patterns of a pattern that is denoted at exactly the same vertices */

   (a) $F := F_m \setminus \{\Gamma_1 \in F | \exists \Gamma_2 \in F, z \in \mathbb{Z} : \Gamma_1 \subset \Gamma_2^{(z)} \wedge \nu(\Gamma_1) = \nu(\Gamma_2)\}$

7. $F_{result} := F$

Part II
List of output files available from ReXSpecies

5 Sequence based figures

One type of figures shows the sequences in an alignment, annotated with transcription factor binding site predictions. As long as they are viewed in ReXSpecies, all elements have pop-ups that show up, if the mouse pointer hovers over them. For the PDFs, this is planned for the future: Hovering a sequence shows up the base pairs under the mouse pointer, hovering a species name shows synonyms, NCBI taxonomic identifier [1], scientific name, and a public domain photograph of an instance of the species. Hovering a prediction is probably the most interesting part. It shows the scores (MSS, BLS) and E-value for the prediction, the sequence logo [2] of the position specific scoring matrix, and, if available, the NCBI gene id [1] of the transcription factor predicted to bind the site. Much more information is shown in that pop-up box, e.g. the set of species and the position specific scoring matrix that was made from these species, but not all is listed here.

The matrix similarity score of the predictions can be coded by the color of the box representing the transcription factor binding site in the alignment image.

The sequence based figures have already been presented in [3].

6 Tree based figures

Another possibility to visualize the results is as a phylogenetic tree with gains and losses annotated at its nodes. This tree image may become very large, because of those annotations, and it is less informative than the combined figures, described below. Thus, we do not plan to support this feature in future versions of ReXSpecies; the software can export PhyloXML[4] to enable the user to use third-party tree visualizers as well. ReXSpecies can show the tree and the alignment using Java applications (ATV[5] and JalView[6]). In the Java applications currently no predictions are shown. To the best of our knowledge, ATV does not support displaying binary characters. For JalView (an alignment visualization tool), we plan to implement the feature in the future.

The tree based figures already have been presented in [3].

7 Combined figures

See also in the main text.

These figures have in common, that all of them include a tree as well as an alignment. The phylogenetic tree is shown left from the alignment, having the leaves replaced by the sequences (the latter is not done for homology based visualizations, see in the main text). If a prediction in the alignment or a pattern at a node is lost, the highlighting boxes get a red border, but by default, losses are not analyzed at all as explained in the Implementation section of the paper.

8 Interactive

Another, more exploratory, sort of figures are generated using JavaScript. This way, the user can explore the alignment (not shown here) and the species tree with its annotations interactively, zooming in certain regions of the tree either as hyperbolic tree [7] or as conventional node link tree diagram. One can browse through this magnified image of the tree showing a few nodes at the same time. See also Figures 5(a), 5(b), 5(c) . To realize this software part, we use a library written by Nicolas Garcia Belmonte called "JavaScript InfoVis Toolkit" ("The JIT").

In the hyperbolic tree figure, all gains and losses for the selected node are listed in a box right from the tree. In the second sort of interactive tree figures, for each node, a small alignment annotated with changes in binding sites along the edge leading to the node is shown. In both figure types, a species image is shown for each node.

The interactive images cannot be exported as as PDF or other formats yet, only screen shots are possible. The generation of interactive PDFs or other interactive file formats is planned for the future.

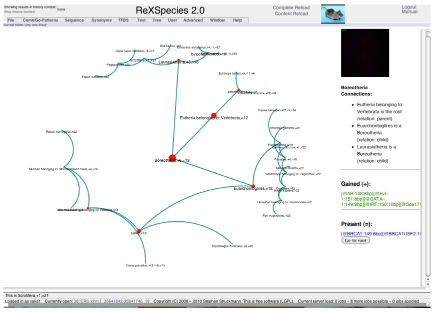

(a) Exploring the hyperbolic tree at the root

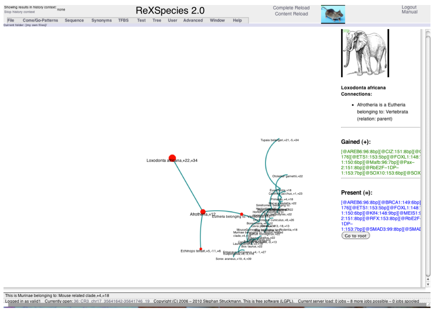

(b) Exploring the hyperbolic tree at a leaf

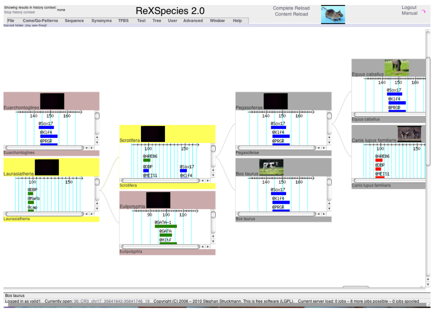

(c) Exploring the node link diagram

Figure 5: Exploring the tree with its annotations interactively using JavaScript

9 Homology based

The homology based figure type is discussed in the main text.

10 As pattern list with tree and pattern logos

The pattern list is discussed in the main text.

11 As BED file

BED file export is discussed in the main text.

References

[1] Sayers EW, Barrett T, Benson DA, Bolton E, Bryant SH, Canese K, Chetvernin V, Church DM, Dicuccio M, Federhen S, Feolo M, Geer LY, Helmberg W, Kapustin Y, Landsman D, Lipman DJ, Lu Z, Madden TL, Madej T, Maglott DR, Marchler-Bauer A, Miller V, Mizrachi I, Ostell J, Panchenko A, Pruitt KD, Schuler GD, Sequeira E, Sherry ST, Shumway M, Sirotkin K, Slotta D, Souvorov A, Starchenko G, Tatusova TA, Wagner L, Wang Y, Wilbur WJ, Yaschenko E, Ye J: **Database resources of the National Center for Biotechnology Information.** *Nucleic Acids Res* 2010, **38**(Database issue):D5–16, [http://dx.doi.org/10.1093/nar/gkp967].

[2] Schneider TD, Stephens RM: **Sequence Logos: A New Way to Display Consensus Sequences**. *Nucleic Acids Res.* 1990, **18**:6097–6100.

[3] Struckmann S, Arauzo-Bravo M, Scholer H, Reinbold R, Fuellen G: **ReXSpecies - a tool for the analysis of the evolution of gene regulation across species**. *BMC Evolutionary Biology* 2008, **8**:111, [http://www.biomedcentral.com/1471-2148/8/111].

[4] Han MV, Zmasek CM: **phyloXML: XML for evolutionary biology and comparative genomics.** *BMC Bioinformatics* 2009, **10**:356, [http://dx.doi.org/10.1186/1471-2105-10-356].

[5] Zmasek CM, Eddy SR: **ATV: display and manipulation of annotated phylogenetic trees**. *Bioinformatics* 2001, **17**:383–384.

[6] Waterhouse AM, Procter JB, Martin DM, Clamp M, Barton GJ: **Jalview Version 2–a multiple sequence alignment editor and analysis workbench**. *Bioinformatics* 2009, **25**:1189–1191.

[7] Lamping J, Rao R, Pirolli P: **A focus+context technique based on hyperbolic geometry for visualizing large hierarchies**. In *CHI '95: Proceedings of the SIGCHI conference on Human factors in computing systems*, New York, NY, USA: ACM Press/Addison-Wesley Publishing Co. 1995:401–408.
