# Supplementary figures and images for: Visualization and Exploration of Conserved Regulatory Modules Using ReXSpecies 2
Source: BMC Evol Biol. 2011 Sep 24;11:267. doi: 10.1186/1471-2148-11-267 (PMC3203875; doi:10.1186/1471-2148-11-267)

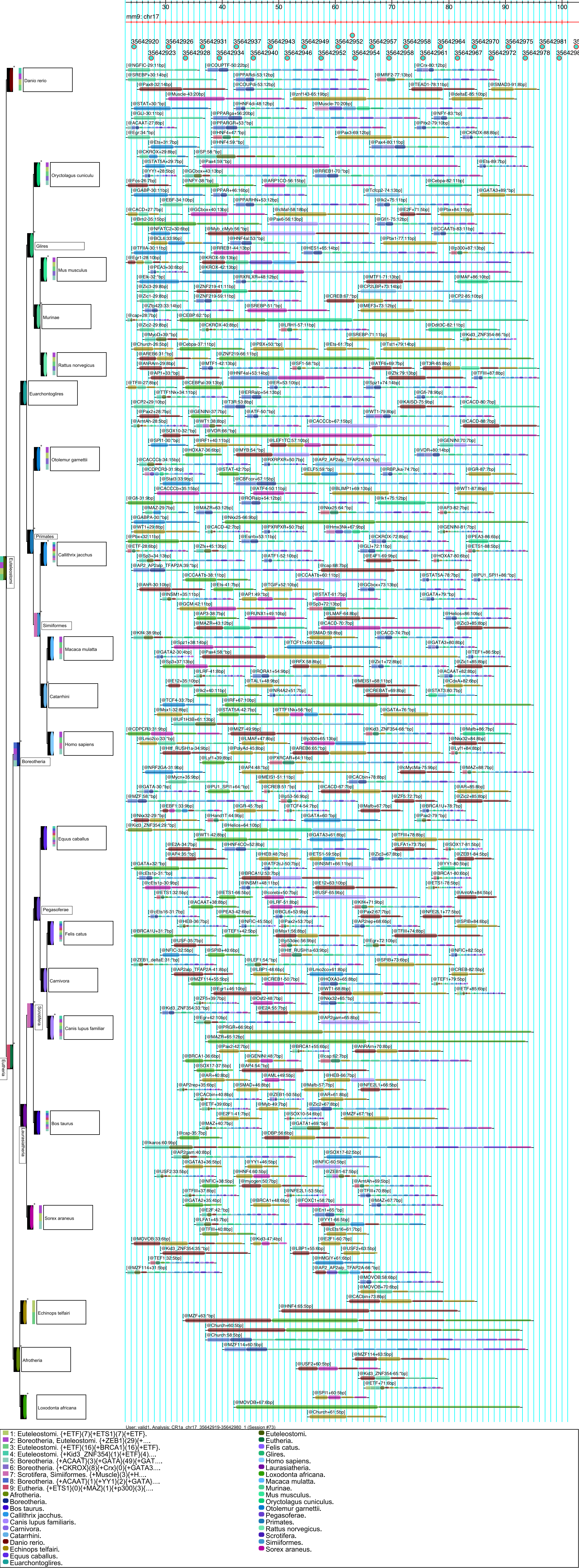

Supplement: Additional file 6 — CR1 patterns, Homology-based figure. The patterns and the transcription factor binding sites found in the murine CR1 region. See Figure 7 for further explanations. [file 1471-2148-11-267-S6.PDF]

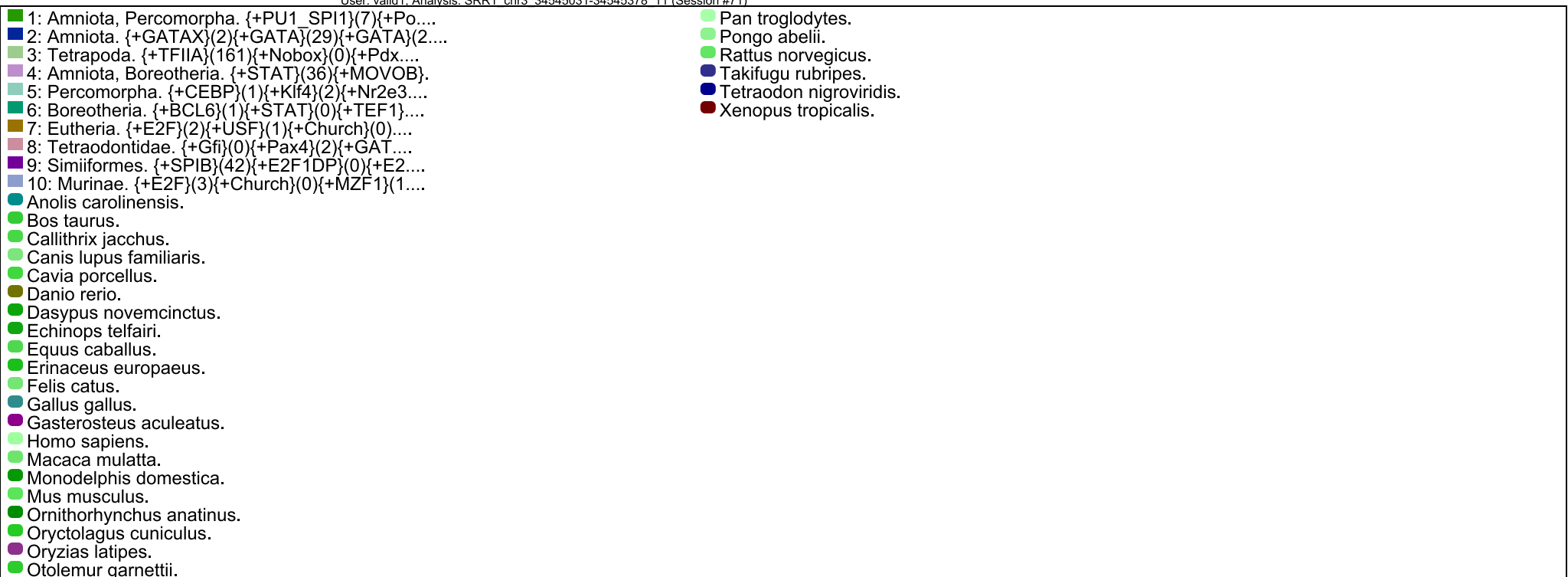

Supplement: Additional file 7 — SRR1 patterns, Homology-based figure. The patterns and the transcription factor binding sites found in the murine SRR1 region. See Figure 7 for further explanations. [file 1471-2148-11-267-S7.PDF]

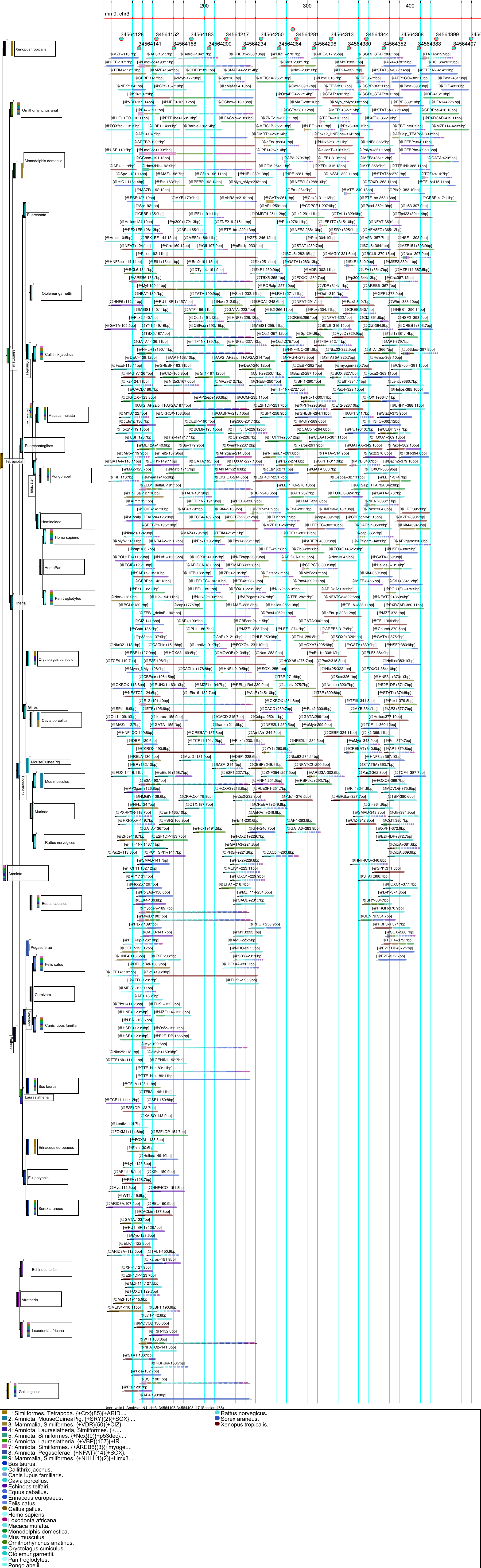

Supplement: Additional file 8 — N1 patterns, Homology-based figure. The patterns and the transcription factor binding sites found in the murine N1 region. See Figure 7 for further explanations. [file 1471-2148-11-267-S8.PDF]

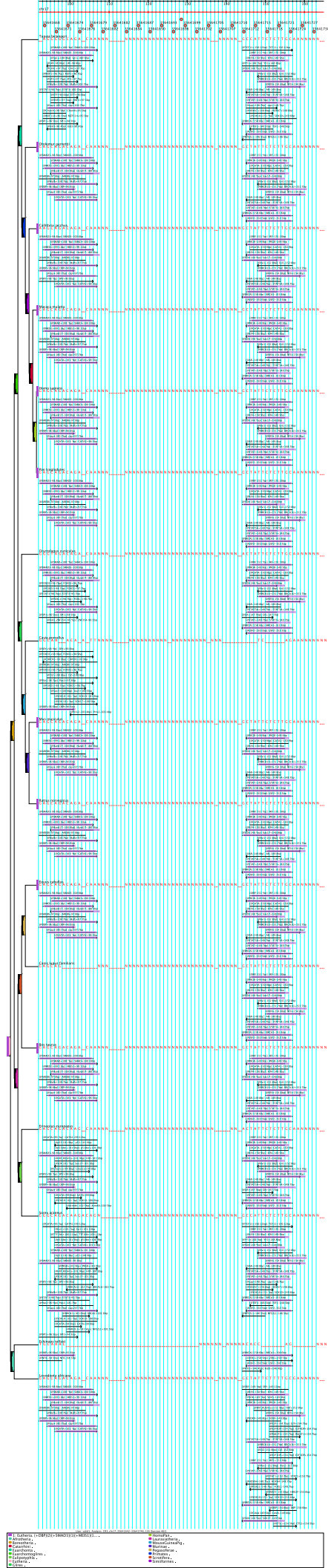

Supplement: Additional file 17 — CR3 pattern, Alignment based figure. The pattern and the transcription factor binding sites found in the murine CR3 region. In principle, the elements of this figure correspond to those in Figure 7. Obviously, the alignment-based figures are much larger than their homology-based version. In contrast to the homology-based figure type, the leaves of the species tree on the left are corresponding to the sequences of the extant species on the right. The sequences are shown in a sequence alignment and the transcription factor binding site predictions are plotted below the sequences. Because the species belonging to each prediction is obviously determined by its vertical position (the leaves are not color coded here), only the thicker part of the colored overlays (see Figure 7) is informative and thus it is shown; it denotes the pattern by referring to its color. To keep the figures readable, by default only the first 10 patterns (ordered by branch length score) are shown in the alignment-based figures. [file 1471-2148-11-267-S17.PDF]

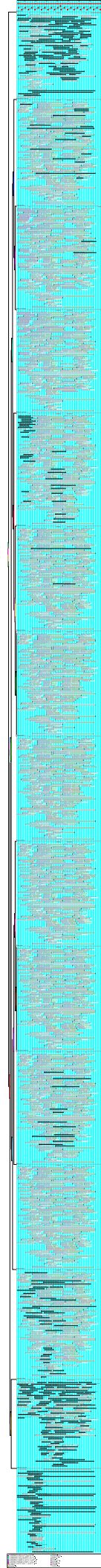

Supplement: Additional file 18 — CR1 patterns, Alignment based figure. The patterns and the transcription factor binding sites found in the murine CR1 region. See Additional File 17 for an explanation. [file 1471-2148-11-267-S18.PDF]

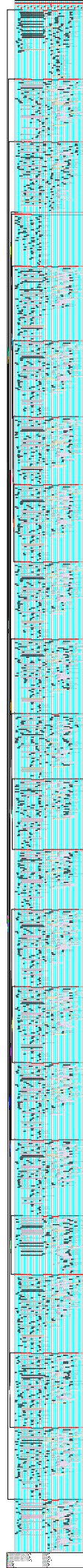

Supplement: Additional file 20 — N1 patterns, Alignment based figure. The patterns and the transcription factor binding sites found in the murine N1 region. See Additional File 17 for an explanation. [file 1471-2148-11-267-S20.PDF]
