# Supplementary material for: Visualization and Exploration of Conserved Regulatory Modules Using ReXSpecies 2
Source: BMC Evol Biol. 2011 Sep 24;11:267. doi: 10.1186/1471-2148-11-267 (PMC3203875; doi:10.1186/1471-2148-11-267)

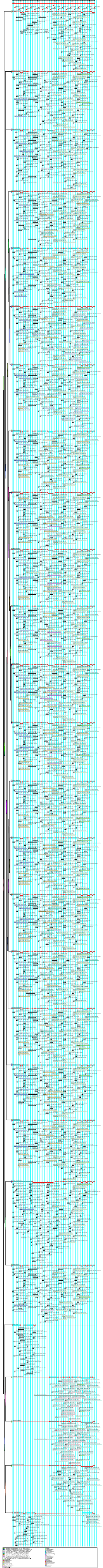

Supplement: Additional file 19 — SRR1 patterns, Alignment based figure. The patterns and the transcription factor binding sites found in the murine SRR1 region. See Additional File 17 for an explanation. [file 1471-2148-11-267-S19.PDF]
